# Functional Specialization of Duplicated *AGAMOUS* Homologs in Regulating Floral Organ Development of *Medicago truncatula*

**DOI:** 10.3389/fpls.2018.00854

**Published:** 2018-07-31

**Authors:** Butuo Zhu, Hui Li, Jiangqi Wen, Kirankumar S. Mysore, Xianbing Wang, Yanxi Pei, Lifang Niu, Hao Lin

**Affiliations:** ^1^Biotechnology Research Institute, Chinese Academy of Agricultural Sciences, Beijing, China; ^2^College of Biological Sciences, China Agricultural University, Beijing, China; ^3^College of Life Sciences, Shanxi University, Taiyuan, China; ^4^Noble Research Institute, LLC, Ardmore, OK, United States

**Keywords:** *AGAMOUS* homologs, C function genes, floral morphogenesis, functional diversification, *Medicago truncatula*, Papilionoideae

## Abstract

The C function gene *AGAMOUS* (*AG*) encodes for a MADS-box transcription factor required for floral organ identity and floral meristem (FM) determinacy in angiosperms. Unlike Arabidopsis, most legume plants possess two *AG* homologs arose by an ancient genome duplication event. Recently, two *euAGAMOUS* genes, *MtAGa* and *MtAGb*, were characterized and shown to fulfill the C function activity in the model legume *Medicago truncatula*. Here, we reported the isolation and characterization of a new *mtaga* allele by screening the *Medicago Tnt1* insertion mutant collection. We found that *MtAGa* was not only required for controlling the stamen and carpel identity but also affected pod and seed development. Genetic analysis indicated that *MtAGa* and *MtAGb* redundantly control *Medicago* floral organ identity, but have minimal distinct functions in regulating stamen and carpel development in a dose-dependent manner. Interestingly, the stamens and carpels are mostly converted to numerous vexillum-like petals in the double mutant of *mtaga mtagb*, which is distinguished from Arabidopsis *ag*. Further qRT-PCR analysis in different *mtag* mutants revealed that *MtAGa* and *MtAGb* can repress the expression of putative A and B function genes as well as *MtWUS*, but promote putative D function genes expression in *M. truncatula*. In addition, we found that the abnormal dorsal petal phenotype observed in the *mtaga mtagb* double mutant is associated with the upregulation of *CYCLOIDEA* (*CYC*)-like TCP genes. Taken together, our data suggest that the redundant *MtAGa* and *MtAGb* genes of *M. truncatula* employ a conserved mechanism of action similar to Arabidopsis in determining floral organ identity and FM determinacy but may have evolved distinct function in regulating floral symmetry by coordinating with specific floral dorsoventral identity factors.

## Introduction

As the important reproductive organs in flowering plants, flowers show remarkable variation in formation and elaboration, and provide the most trustworthy external characteristics for establishing relationships among different angiosperm species. In dicots, flowers are commonly composed of four different types of floral organs arranged in concentric whorls. From outside to the center, these floral organs are sepals in the first whorl, petals in the second whorl, stamens in the third whorl and carpels in the fourth whorl. Understanding how these distinct floral organs are specified has been a long-standing question in plant development and held the fascination of scientists for centuries (Meyerowitz et al., 1989; Schwarz-Sommer et al., 1990; Coen and Meyerowitz, 1991; Theissen et al., 2016).

In the past two decades, extensive genetic and molecular analyses of a series of floral homeotic mutants in diverse species have revealed that floral organ formation is controlled by several conserved floral organ identity genes and these floral organ regulators were proposed to function in simple genetic models. Based on the studies in model dicot plants *Arabidopsis thaliana* and *Antirrhinum majus*, the most well-known “ABC” model was outlined (Schwarz-Sommer et al., 1990; Bowman et al., 1991; Coen and Meyerowitz, 1991). In Arabidopsis, the A function genes *APETALA1* (*AP1*) (Mandel et al., 1992) and *APETALA2* (*AP2*) (Jofuku et al., 1994) alone determine the identity of sepals in the first whorl. However, the combined activity of A function genes and B function genes including *APETALA3* (*AP3*) (Jack et al., 1992) and *PISTILLATA* (*PI*) (Goto and Meyerowitz, 1994) is required for the formation of petals in the second whorl. Establishment of the stamen identity in the third whorl is controlled by B function genes and C function gene *AGAMOUS* (*AG*) (Yanofsky et al., 1990), whereas the C function gene *AG* solely determinates the termination and differentiation of the floral meristem (FM) into carpels (Wellmer et al., 2014). Later studies revealed that more genes are involved in regulating ovule identity and development inside the carpel (Angenent et al., 1995; Colombo et al., 1995). In Arabidopsis, three MADS-domain family members *SHATTERPROOF1* (*SHP1*, formerly known as *AGL1*), *SHATTERPROOF2* (*SHP2*, formerly known as *AGL5*) and *SEEDSTICK* (*STK*, formerly known as *AGL11*) have been identified as D function genes and the *shp1 shp2 stk* triple mutant convert ovules into carpel-like or leaf-like structures (Favaro et al., 2003; Pinyopich et al., 2003). In addition, an E function has been assigned to another class of genes, including *SEPALLATA1* (*SEP1*, formerly known as *AGL2*), *SEP2* (*AGL4), SEP3* (*AGL9*), and *SEP4* (*AGL3*), which are essential for the identity of all floral organs in combination with the A, B, C, and D function genes (Pelaz et al., 2000; Ditta et al., 2004). The expanded “ABCDE” model maintains organ identity by a refined combination as that A+E genes control sepals, A+B+E genes specify petals, B+C+E genes determine stamens, C+E genes control carpels and C+D+E genes specify ovules (Theissen et al., 2016).

In contrast to the flowers of model dicot species such as *A. thaliana* or *A. majus*, the zygomorphic flowers in Papilionoideae plants are peculiarly arranged with pentamerous whorls of sepals and petals, two whorls of five stamens each, and a single carpel (Tucker, 2003). To investigate the molecular basis of floral organ identity in Papilionoideae, several floral organ regulation genes have been identified and characterized in the model legume *Medicago truncatula*. The *MtPIM* gene, a homolog of the A function gene *AP1* in *M. truncatula*, is required for specification of floral meristem identity. Mutation of *MtPIM* leads to a flower-to-inflorescence conversion and altered flowers with sepals transformed into leaves, indicating that *MtPIM* controls FM identity and flower development (Benlloch et al., 2006). Function analysis of the *MtNMH7* and *MtTM6*, which are homologs of the Arabidopsis B function gene *AP3*, revealed that *MtNMH7* appears to play a major role in determining the petal identity, whereas *MtTM6* plays a more important role in the stamen identity (Roque et al., 2013). Both *MtPI* and *MtNGL9* encode MADS-box transcription factors related to the *Arabidopsis PISTILLATA* gene. Mutation of *MtPI* leads to defects in petals and stamens, suggesting that *MtPI* functions as a master regulator of B-function in *M. truncatula* (Benlloch et al., 2009; Roque et al., 2016). Although the floral organ arrangement and flower morphogenesis between Arabidopsis and *M. truncatula* are apparently different, the fact that orthologs of *AP1, AP3* and *PI* have also been identified in *M. truncatula* suggests that the “ABCDE” model is generally applicable to Papilionoideae plants as well.

In Arabidopsis and Antirrhinum, the C function is, respectively, represented by *AG* and *PLENA* (*PLE*), which is required to control the stamen, carpel, ovule identity, to prevent the mis-expression of A function genes in the third and fourth whorls, and to establish the FM determinacy by antagonizing the key regulator of stem cell homeostasis *WUSCHEL* (*WUS*) (Bowman et al., 1989, 1991; Yanofsky et al., 1990; Bradley et al., 1993; Lenhard et al., 2001). By contrast, due to an ancient genome duplication event, the presence of duplicated *AG* homologs has been commonly found in several Papilionoideae plants including *Lotus japonicus, Pisum sativum*, and soybean (Dong et al., 2005; Shoemaker et al., 2006; Serwatowska et al., 2014). Recently, two *AGAMOUS* homologs *MtAGa* and *MtAGb* were functionally characterized and shown to fulfill the C function activity that promote complete stamen and carpel identity and FM determinacy in *M. truncatula* (Serwatowska et al., 2014). However, neither *mtaga* and *mtagb* single mutant nor VIGS/RNAi lines silenced *MtAGa* and *MtAGb* both show a complete loss of C-function phenotype as observed in Arabidopsis *ag* mutant (Serwatowska et al., 2014). Moreover, how the duplicated *AGAMOUS* homologs interact with other floral organ identity genes during *M. truncatula* floral morphogenesis remains to be elucidated.

In this study, we reported the isolation and characterization of a new *mtaga* allele by screening *Tnt1* retrotransposon-tagged lines of *M. truncatula*. We found that *MtAGa* was not only required for controlling the stamen and carpel identity, but also affected pod and seed development. The *mtaga mtagb* double mutant analysis confirmed that *MtAGa* and its paralog *MtAGb* together fulfill a full C-function activity but exhibit minimal subfunctionalization in regulating stamen and carpel identity. Further comprehensive molecular analysis revealed that the duplicated *AGAMOUS* homologs *MtAGa* and *MtAGb* coordinate with floral dorsoventral identity regulators to regulate flower morphogenesis in *M. truncatula.*

## Materials and Methods

### Plant Materials and Growth Conditions

Wild-type *M. truncatula* ecotype R108 and mutants were grown in greenhouse with 25°C/16-h (day) and 23°C/8-h (night) photoperiods at 60–70% humidity and 150–200 μmol m^2^s light intensity.

### Mutant Screening and Gene Cloning

Insertional mutagenesis in *M. truncatula* genotype R108 using *Tnt1* retrotransposon and screening conditions in the greenhouse was carried out as previously described (Tadege et al., 2008; Yarce et al., 2013). Forward genetics screening of *Tnt1*-tagged lines under standard conditions (16 h/8 h and 24°C/20°C day/night cycles) in greenhouse for flower mutants led to identification of the mutant line NF19601 with split carpels. To identify the gene linked to the split-carpel phenotype, *Tnt1* flanking sequences of NF19601 mutant were recovered using TAIL-PCR (Cheng et al., 2014) and genotyped by PCR using flanking sequence tag (FST)-specific primers (Supplementary Table S1). One FST segregated with the homozygous mutant was analyzed by BLAST search against the *M. truncatula* genome at the National Center for Biotechnology Information (NCBI)^1^ and Phytozome^2^. Reverse screening of *Tnt1* insertion lines were performed following the standard screening protocol (Cheng et al., 2014).

### Sequence Alignment and Phylogenetic Analysis

Amino acid sequences of *M. truncatula* and other selected species were aligned using ClustalW^3^, and a neighbor-joining phylogenetic tree was constructed based on the amino acid sequences of the full-length proteins using the MEGA 6 software. The most parsimonious tree with bootstrap values from 1,000 trials was used. Accession numbers used in this study were listed in Supplementary Table S2.

### RNA Extraction and Quantitative Real-Time PCR (qRT-PCR) Analysis

Total RNA from different *M. truncatula* tissues was extracted using the Trizol method, and RNA was treated with Turbo DNase (Ambion) to remove genomic DNA. Five micrograms of total RNA was used for cDNA synthesis by using the SuperScript^®^ III First-Strand Synthesis System for RT-PCR kit (Life Technologies). qRT-PCR was performed using the ROCHE LightCycler^®^ 96 detect system with the TransStart Tip Green qPCR SuperMix (TransGen Biotech). qRT-PCR data were obtained using three biological replicates and transcripts were normalized to *MtActin*. Primers used were listed in Supplementary Table S1.

### Subcellular Localization Analysis

The coding sequences of *MtAGa* and *MtAGb* were PCR amplified from wild-type *M. truncatula* ecotype R108 and cloned into the pENTR/D-TOPO cloning vector (Invitrogen), then transferred into the *pMDC83* Gateway vector using the Gateway LR reaction (Invitrogen) to generate the *pMDC83-MtAGa-GFP* and *pMDC83-MtAGb-GFP* destination vectors. The constructs were introduced into *A. tumefaciens* stain GV2260 by chemical transformation and the agrobacterium was infiltrated into 4-week-old *Nicotiana benthamiana* leaves. P19 was used to inhibit transgenic silencing. After culturing for 2–3 days, the GFP signal was visualized under the Zeiss LSM700 confocal laser-scanning microscope. Primers used for vectors construction were listed in Supplementary Table S1.

### Histological Analysis

Juvenile flowers of *M. truncatula* from 3-month-old plants were fixed and embedded as previously described (Lin et al., 2009). The tissues were sliced into 8- to 10-μm sections with a Leica RM2265 microtome, affixed to microscope slides, and stained with Toluidine blue. Images were obtained with a digital camera mounted on the Olympus BX-51compound microscope.

### Scanning Electron Microscopy (SEM)

For SEM, fresh *M. truncatula* floral buds from 5-month-old wild-type and different mutant plants were fixed by vacuum infiltration with 3.0% glutaraldehyde in 25 mM phosphate buffer (pH 7.0) for 2 days, then plant tissues were further fixed with 1.0% osmium tetroxide in the same phosphate buffer for 2 h and subsequently dehydrated in a graded ethanol series. The desiccated tissues were critical-point dried in liquid CO_2_, mounted on aluminum stubs, and sputter coated with gold. Specimens were then observed using a JSM-8404 microscope (S3400N; Hitachi Ltd.).

## Results

### Identification and Characterization of a New *mtaga* Allele in *M. truncatula*

**FIGURE 1:**
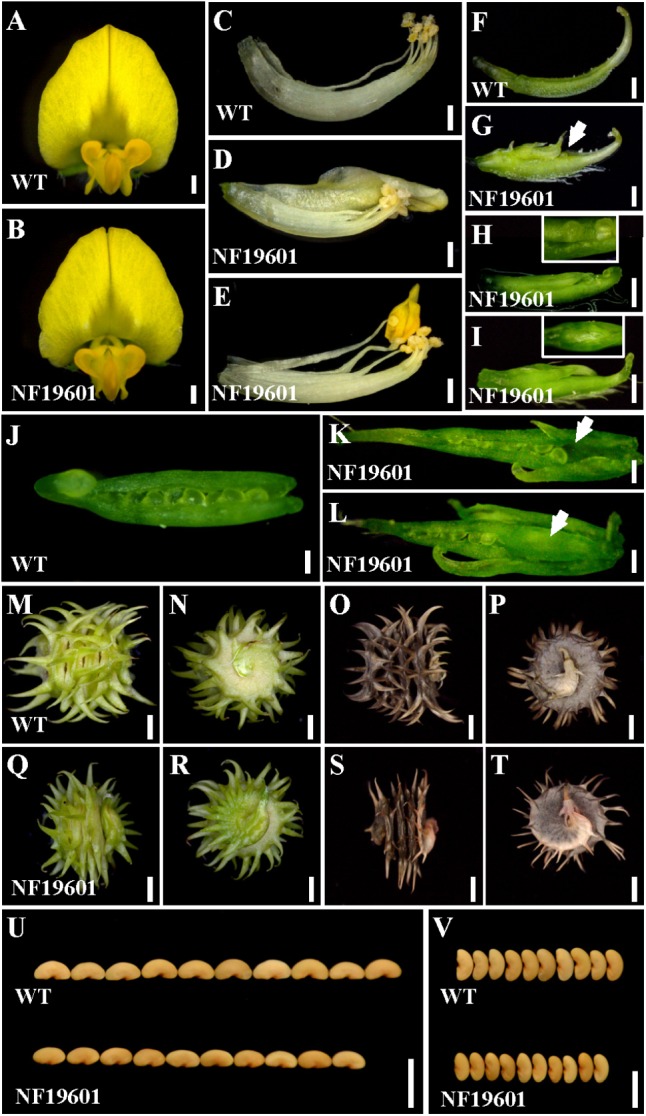
NF19601 mutant of *M. truncatula* shows defects in floral organ, pod and seed development. **(A,B)** Flowers of the wild-type and NF19601. Bars = 500 μm. **(C–E)** Stamens of the wild-type and NF19601. The staminal tube is slightly separated in NF19601. Bars = 500 μm. **(F–I)** Comparison of carpels in the wild-type and NF19601. White arrow in **(G)** points to the exposed ovule. The inset in **(H)** shows a close-up view of the distal part of the carpel with exposed ovules. The inset in **(I)** shows a ventral view of the paraxial part of the carpel with a floral bud-like structure formed. Bars = 500 μm. **(J**–**L)** Dissected carpels of the wild-type and NF19601. White arrow in **(K)** shows no ovules in the carpel base of NF19601. White arrow in **(L)** shows a floral bud-like structure in the carpel base of NF19601. Bars = 200 μm. **(M–P)** Side **(M,O)** and bottom **(N,P)** views of the wild-type immature **(M,N)** and mature **(O,P)** pods. Bars = 2 mm. **(Q–T)** Side **(Q,S)** and bottom **(R,T)** views of NF19601 immature **(Q,R)** and mature **(S,T)** pods. Bars = 2 mm. **(U,V)** Comparison of seed length **(U)** and width **(V)** between the wild type and NF19601. Bars = 5 mm.

One floral development mutant NF19601 was identified from a forward genetic screening of *Tnt1* retrotransposon-tagged population in *M. truncatula* genotype R108 (Tadege et al., 2008; Yarce et al., 2013). In contrast to the wild-type, the mutant flowers were morphologically normal in the sepals and petals (**Figures 1A,B**), whereas the staminal tube was slightly separated and the stamens were occasionally transformed into petal-like structures (**Figures 1C–E**). Besides, multiple carpels were frequently observed and trichomes were formed on the back of the main carpel in NF19601 (**Figures 1F–I**). A close examination of the carpels showed that the mutant had exposed ovules and some ovules were missing in the basal part of carpels (**Figures 1H,J–L**). Sometimes floral bud-like structures were formed at the bottom of carpels (**Figures 1I,L**). In this mutant, the defective carpels developed to abnormally spiny pods with reduced whirl numbers (**Figures 1M–T** and Supplementary Figure S1A), subsequently resulting in decreased seed number per pod (Supplementary Figure S1B). In addition, the seed size and weight in the floral mutant also significantly decreased compared to that of wild-type (**Figures 1U,V** and Supplementary Figures S1C,D). Taken together, these phenotypes indicated that this mutant is defective in the floral organ identity, especially in the development of stamens and carpels in *M. truncatula*.

**FIGURE 2:**
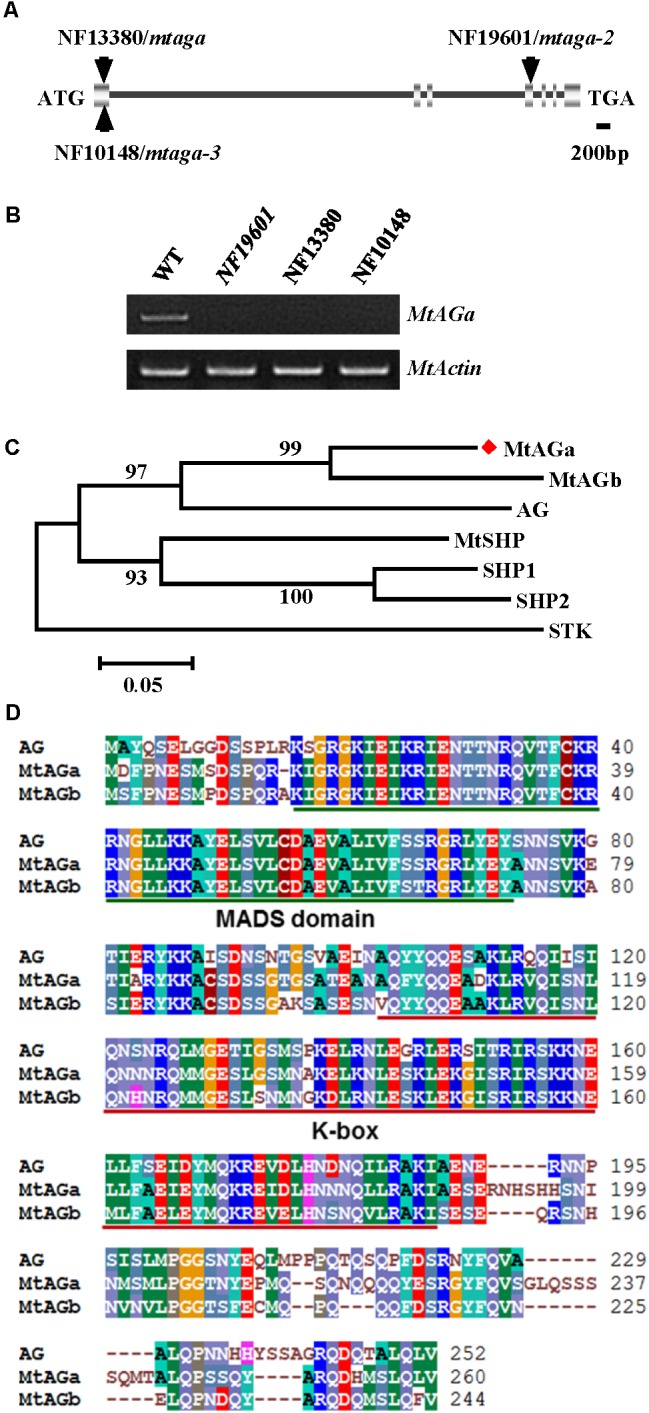
Molecular cloning of *MtAGa* in *M. truncatula*. **(A)** Schematic representation of the gene structure of *MtAGa* and the *Tnt1* insertion sites of NF19601, NF13380, and NF10148. **(B)** RT-PCR analysis of *MtAGa* expression in young flowers of the wild-type, NF19601, NF13380, and NF10148. *MtActin* was used as the loading control. **(C)** Phylogenetic analysis of *MtAGa* and its homologs in *M. truncatula* and Arabidopsis based on the amino acid sequence of the full-length protein. Numbers on branches indicate bootstrap values for 1,000 replicates. **(D)** Sequence alignment of MtAGa, MtAGb, and Arabidopsis AG. The conserved MADS domain and K-box were highlighted with color lines, respectively.

To identify the gene associated with the mutant phenotype, thermal asymmetric interlaced-PCR was performed to recover the flanking sequences of *Tnt1* from line NF19601 (Tadege et al., 2008). Based on the genotyping results, one *Tnt1* insertion segregating with the mutant phenotype was identified. Genetic analysis revealed that the mutant phenotype segregates as a single-gene recessive mutant and all mutant plants harbored homozygous insertion for the particular FST. The full length gene sequence corresponding to this FST was recovered and sequence alignment showed that the candidate gene encodes a MADS-box transcription factor, which is identical to previously reported *MtAGa* (Serwatowska et al., 2014). Genomic PCR analysis showed that the *Tnt1* was inserted in the fourth exon of *MtAGa*, resulting in the abolished transcription of the full-length *MtAGa* (**Figures 2A,B**). To confirm that the phenotypes of defective floral organ development as well as abnormal pods and seeds were caused by disruption of *MtAGa*, two additional *Tnt1* insertion lines NF13380 (*mtaga/mtag-2*) and NF10148 (named *mtaga-3* here) were obtained by reverse screening (Cheng et al., 2014; Serwatowska et al., 2014; **Figures 2A,B**). Phenotypic observation showed that both NF10148 and NF13380 mutants displayed same phenotypes as described in NF19601 (named *mtaga-2* here), confirming that the *MtAGa* gene is not only required for controlling stamen, carpel identity and FM determinacy, but also functions in pod and seed development in *M. truncatula.*

### Functional Specialization of *MtAGa* and *MtAGb* in Regulating Floral Organ Identity, Pod Shape, and Seed Size

**FIGURE 3:**
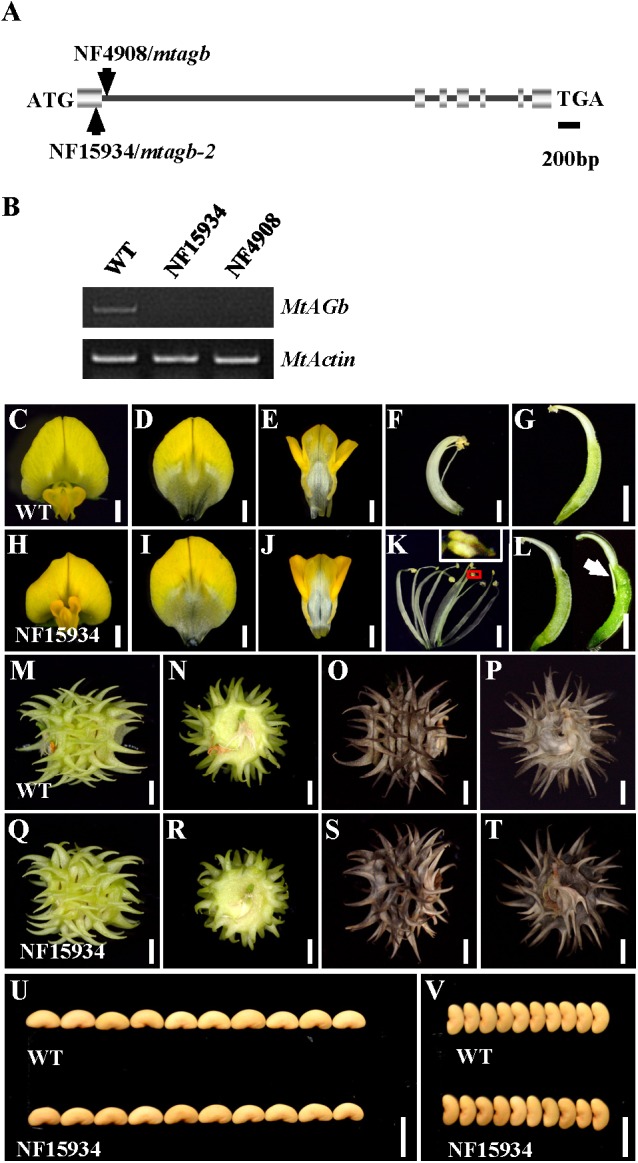
Characterization of the *mtagb* mutant. **(A)** Schematic representation of the gene structure of *MtAGb* and the *Tnt1* insertion sites of NF15934 and NF4908 are shown. **(B)** RT-PCR showed the expression level of *MtAGb* in young flowers of the wild-type, NF15934 and NF4908. *MtActin* was used as the loading control. **(C,H)** Flowers of the wild-type and NF15934. Bars = 1 mm. **(D–G)** Dissected flower of the wild-type. The top view of vexillum **(D)**, fused alae and keel petals **(E)**, the side view of stamen **(F)** and carpel **(G)**. Bars = 1 mm. **(I–L)** Dissected flower of NF15934. The top view of vexillum **(I)**, fused alae and keel petals **(J)**, the side view of stamen **(K)** and carpel **(L)**. The inset in **(K)** shows the tip of anther changed to a petal-like structure. White arrow in **(L)** shows the stigmatic protuberance. Bars = 1 mm. **(M–P)** Side **(M,O)** and bottom **(N,P)** views of the wild-type immature **(M,N)** and mature **(O,P)** pods. Bars = 2 mm. **(Q–T)** Side **(Q,S)** and bottom **(R,T)** views of NF15934 immature **(Q,R)** and mature **(S,T)** pods. Bars = 2 mm. **(U,V)** Comparison of seed length **(U)** and width **(V)** between the wild-type and NF15934. Bars = 5 mm.

In agreement with previous findings that *MtAGa* and *MtAGb* both show C-function activity in regulating *M. truncatula* floral development (Serwatowska et al., 2014), phylogenetic analysis revealed that MtAGa and its homolog MtAGb are grouped in a separate subclade of C-function family (**Figure 2C**) and amino acid sequence alignment indicated that MtAGa, MtAGb and Arabidopsis AG have the highly conserved MADS domain and K-box (**Figure 2D**). To investigate possible roles of *MtAGb* in *M. truncatula* pod and seed development, two *Tnt1* insertion mutants of *MtAGb* were characterized. One mutant is NF4908, which has been identified as *mtagb*, containing a *Tnt1* insertion in the first intron of *MtAGb* (Serwatowska et al., 2014; **Figure 3A**). The other mutant is NF15934 (named *mtagb-2* here), which was newly identified from the *Tnt1* insertional population by BLAST-searching the FST database (Cheng et al., 2014), containing a *Tnt1* insertion in the first exon of *MtAGb* (**Figure 3A**). RT-PCR analysis showed that the expression of full length *MtAGb* were undetectable in both two alleles (**Figure 3B**). Consistent to the previous report, both NF15934 and NF4908 mutants exhibited normal sepals and petals, but showed an incomplete fusion of stamen tubes and weak petaloid structures developed at stamen tips (**Figures 3C–F,H–K**). Moreover, compared to wild-type and *mtaga*, the *mtagb* mutants showed a weak developmental defect in carpels with occasionally split carpels but no extra structure formation at the bottom (**Figures 3G,L**), and the pods and seeds in *mtagb* mutants were nearly indistinguishable from the wild-type (**Figures 3M–V** and Supplementary Figure S2). These data suggest that *MtAGb* appears to play a major role in controlling stamen fusion, whereas *MtAGa* contributes more to carpel development.

### Comparison of Expression Pattern and Subcellular Localization of MtAGa and MtAGb

**FIGURE 4:**
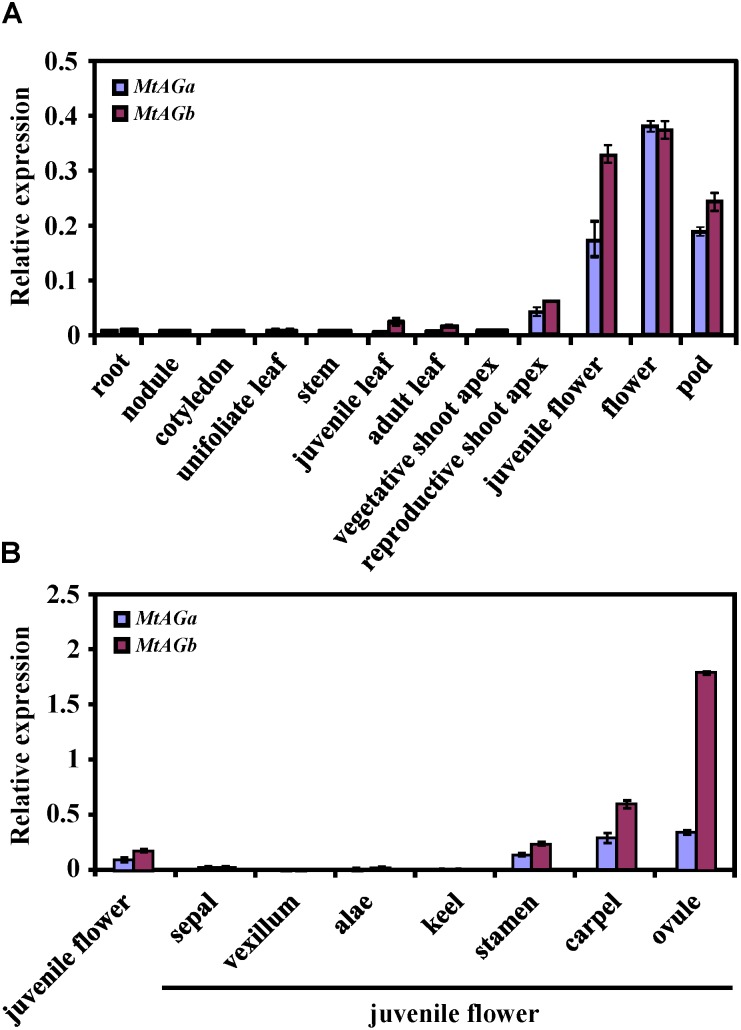
Comparison of expression pattern between *MtAGa* and *MtAGb*. **(A)** *MtAGa* and *MtAGb* expression levels revealed by qRT-PCR in different tissues. Values are means ± SE of three biological replicates. **(B)** *MtAGa* and *MtAGb* expression levels revealed by qRT-PCR analysis in different floral organs. The flower organs were dissected from juvenile flowers. Values are means ± SE of three biological replicates.

To better understand the functional specialization of *MtAGa* and *MtAGb* in controlling reproductive organ’s determination, we analyzed the expression patterns of *MtAGa* and *MtAGb* in different tissues and organs. qRT-PCR analysis revealed that both *MtAGa* and *MtAGb* are highly expressed in flowers and pods, medially expressed in floral apices, and lowly expressed in other tissues (leaf, stem, cotyledon, root, and nodule) (**Figure 4A**). Further qRT-PCR analysis of dissected floral organs revealed that both *MtAGa* and *MtAGb* are predominantly expressed in stamens, carpels as well as ovules with different expression levels (**Figure 4B**). This tissue-specific expression pattern is consistent with the proposed scenario of *MtAGa* and *MtAGb* fulfilling a C function activity during floral development in *M. truncatula*.

To determine the subcellular localization of the MtAGa and MtAGb proteins, we performed a transient expression experiment in leaf epidermal cells of *N. benthamiana*. The C terminus of MtAGa and MtAGb were individually fused with green fluorescent protein (GFP) under the control of Cauliflower mosaic virus (CaMV) 35S promoter. In contrast to the control, which was ubiquitous in leaf epidermal cells, the MtAGa-GFP and MtAGb-GFP fusion proteins were localized in both cytoplasm and nucleus (Supplementary Figure S3).

### Genetic Analysis of *MtAGa* and *MtAGb* in *M. truncatula* Flower Development

**FIGURE 5:**
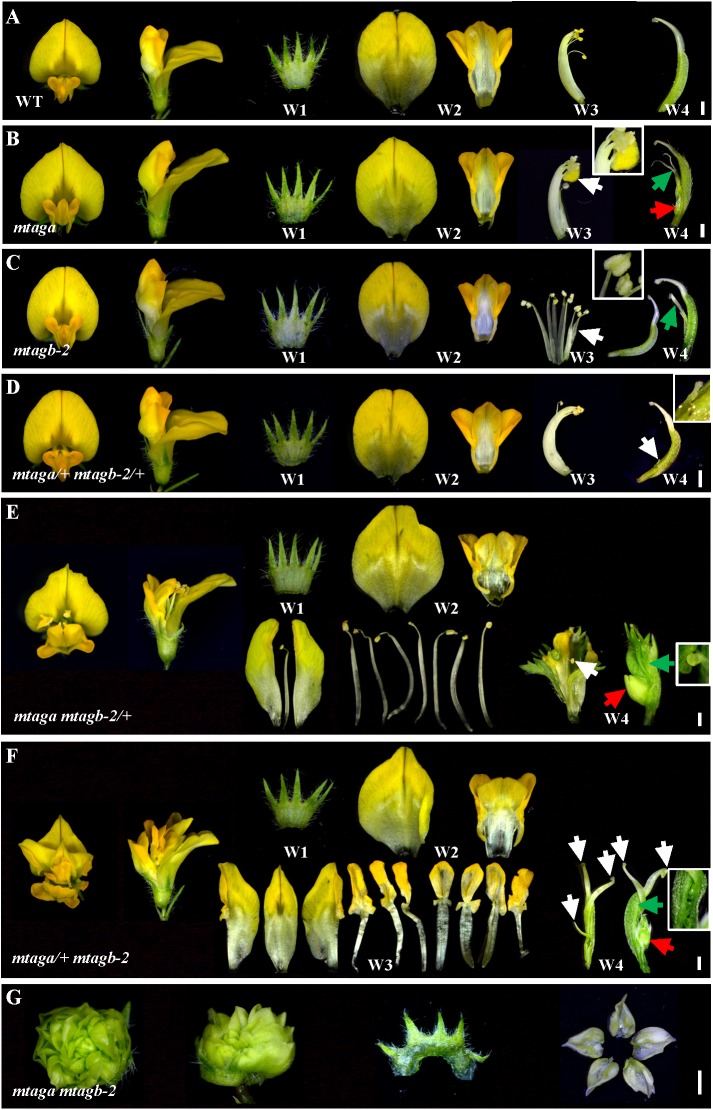
Floral phenotypes of different *mtag* mutants. **(A)** Dissected flowers of the wild-type showing normal four whorls (W1–W4). Bar = 1 mm. **(B)** Dissected flowers of the *mtaga* mutant. The white arrow indicates anther turned into petal-like structure and the inset is magnification of indicated region. The green arrow in W4 points to the split carpels and the red arrow indicates the floral bud-like structure formed at the bottom of the carpel. Bar = 1 mm. **(C)** Dissected flowers of the *mtagb* mutant. The white arrow in W3 indicates the unfused staminal tube. The inset view shows the anther tips which were changed into petal-like structure. Occasionally, the split carpel can be found in W4 (green arrow). Bar = 1 mm. **(D)** Dissected flowers of the *mtaga/+ mtagb-2/+* plants. The floral phenotype of *mtaga/+ mtagb-2/+* is similar to that of the wild-type, but occasionally the split carpel could be found in W4. The white arrow in W4 indicates the filamentous structure and the inset is magnification of indicated region. Bar = 1 mm. **(E)** Dissected flowers of the *mtaga mtagb-2/+* plants. The white arrow indicates the wrapped stamen in the center of the W4, the red arrow shows the floral bud-like structure formed at the base of the carpel. The inset is magnification of indicated region by green arrow showing existed ovules. Bar = 1 mm. **(F)** Dissected flowers of the *mtaga/+ mtagb-2* plants. The white arrows indicate multiple carpels, red arrow shows the floral bud-like structure at the carpel base. The inset is magnification of indicated region by green arrow showing existed ovules. Bar = 1 mm. **(G)** Dissected flowers of the *mtaga mtagb-2* double mutant. The W2–W4 whorls mostly changed into vexillum-like petals. Bar = 1 mm.

**FIGURE 6:**
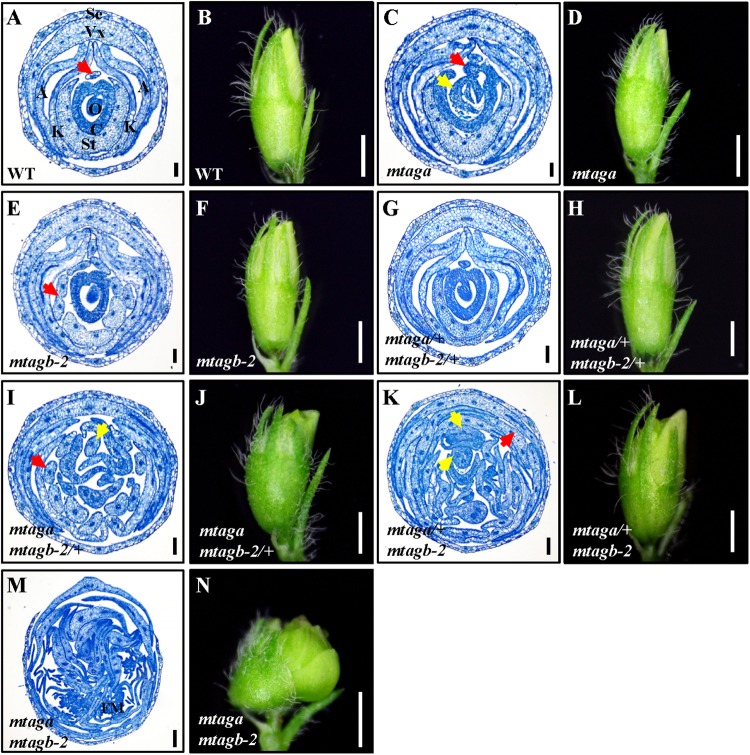
The histological analysis of juvenile flowers in the wild-type and different *mtag* mutants. **(A,B)** Cross section and morphology of juvenile flower in the wild-type. The red arrow points to the stamen filament separated from the fused nine. Se, sepal; Vx, vexillum; A, alae; K, keel; St, staminal tube; C, carpel; O, ovule. Bar = 100 μm in **(A)**, 1 mm in **(B)**. **(C,D)** Cross section and morphology of juvenile flower in *mtaga* mutant. Red arrow indicates exposed ovule and yellow arrow points to the extra floral bud-like structure, respectively. Bar = 100 μm in **(C)**, 1 mm in **(D)**. **(E,F)** Cross section and morphology of juvenile flower in *mtagb* mutant. The red arrow indicates the incompletely fused staminal tube. Bar = 100 μm in **(E)**, 1 mm in **(F)**. **(G,H)** Cross section and morphology of juvenile flower in *mtaga/+ mtagb-2/+.* Bar = 100 μm in **(G)**, 1 mm in **(H)**. **(I,J)** Cross section and morphology of juvenile flower in *mtaga mtagb-2/+.* The red arrow indicates the separated staminal tube, the yellow arrow points to the extra stamen. Bar = 100 μm in **(I)**, 1 mm in **(J)**. **(K,L)** Cross section and morphology of juvenile flower in *mtaga/+ mtagb-2.* Red arrow points to petaloid organ and yellow arrows indicate the extra floral bud-like structures. Bar = 100 μm in **(K)**, 1 mm in **(L)**. **(M,N)** Cross section and morphology of juvenile flower in the *mtaga mtagb-2* double mutant. FM, floral meristem. Bar = 100 μm in **(M)**, 1 mm in **(N)**.

In Arabidopsis, *AGAMOUS* is the only C function gene, which is required for stamen and carpel identity and FM determinacy. Mutation of *AG* in Arabidopsis causes lack of pistils and stamens, and the flowers continue to produce sepals and petals (Bowman et al., 1989). However, in *M. truncatula*, either *mtaga* and *mtagb* single mutants or VIGS/RNAi lines silenced *MtAGa* and *MtAGb* did not show a complete loss of C-function phenotype as observed in Arabidopsis *ag* mutant (Serwatowska et al., 2014). To explore whether *MtAGa* and *MtAGb* fulfill a full C-function activity, we generated the *mtaga mtagb-2* double mutant by crossing NF13380 and NF15934 and the resulting double mutant was analyzed for morphological phenotypes. Compared with wild-type and single mutants, the *mtaga mtagb-2* double mutant showed additive effect on stamen and carpel development (**Figures 5**, **6**). In the *mtaga* flower, the central carpel was frequently separated and was not able to fully enclose the ovules (**Figures 5B**, **6C**), while in the *mtagb-2* flower, the staminal tube was poorly fused and the stamen tips were frequently transformed into petal-like structures (**Figures 5C**, **6E**). However, in the *mtaga mtagb-2* double mutant, the stamens and carpels were mostly converted to numerous vexillum-like petals, which may be derived from multiple FMs (**Figures 5G**, **6M,N**). These results clearly indicate that *MtAGa* and *MtAGb* together fulfill a full C-function activity but also suggest that *MtAGa* and *MtAGb* may affect petal shape somehow.

Interestingly, during the screening of the *mtaga mtagb-2* double mutant, we found that some of the F2 progeny from the NF13380 × NF15934 crossing showed peculiar floral phenotypes, which are different from the single and double mutants. Genotyping analysis revealed that these peculiar floral phenotypes are associated with particular genotypes. Compared with the wild-type and single mutants, the *mtaga mtagb-2/+* (*/+* means the heterozygous mutant) flowers displayed normal sepals and petals in whorl 1 and 2, but in whorl 3 stamens usually changed into irregular petals or petaloid structures, and extra organs such as filament-like structure and petaloid extensions were often observed (**Figures 5E**, **6I** and Supplementary Figure S4A). Whorl 4 was usually composed of mosaic organs of sepaloid and petaloid structures and/or leaf-like structures in place of carpels and ovules (**Figures 5E**, **6I** and Supplementary Figures S4B,C). Moreover, a new layer of floral organs like stamen or floral bud were frequently formed in the center of whorl 4, suggesting FM determinacy was disrupted in *mtaga mtagb-2/+* (**Figures 5E**, **6I** and Supplementary Figure S4B). By contrast, in the *mtaga/+ mtagb* plants, flowers also displayed normal whorl 1 and 2, but in whorl 3, three of 10 stamens were usually converted to one vexillum and two alae-like petals, the other seven stamens showed petaloid structures (**Figures 5F**, **6K**). Besides, extra petal and petaloid tissues were often observed in whorl 3 (**Figure 6K** and Supplementary Figure S5A). Whorl 4 was frequently composed of multiple carpels similar to *mtaga*, but leaf-like tissues and new floral buds were often found in the center of whorl 4 (**Figures 5F**, **6K** and Supplementary Figure S5B), suggesting *mtaga/+ mtagb-2* mutant flowers were indeterminate as well. However, in double heterozygote *mtaga/+ mtagb-2/+* plants, whorl 1–3 of flowers did not exhibit any obvious phenotypic defects compared to that of wild-type (**Figures 5A,D**, **6A,G**), but in whorl 4, a filamentous structure was observed at the carpel base (**Figure 5D**). These observations indicated that *MtAGa* and *MtAGb* redundantly control floral organs identity in whorl 3 and 4 of *M. truncatula*, but have minimal distinct functions in regulating stamen and carpel development in a dose-dependent manner.

### Scanning Electron Microscopy (SEM) Analysis of Floral Development in *mtag* Mutants

**FIGURE 7:**
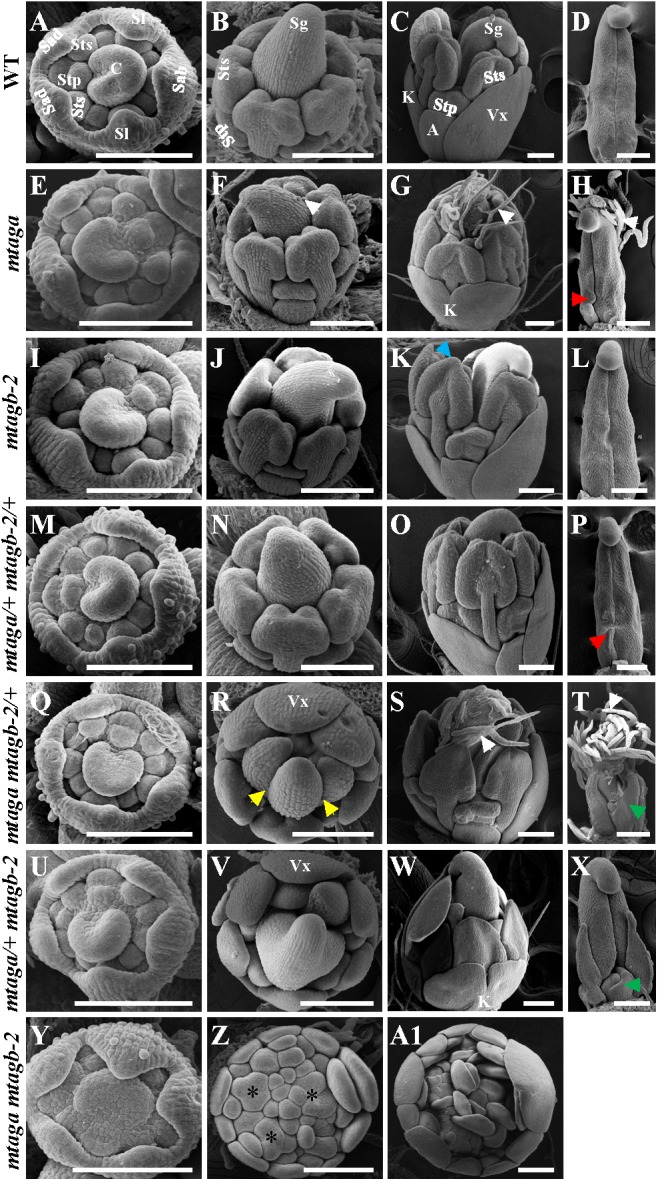
The scanning electron microscopy (SEM) analysis of floral development in the wild-type and *mtag* mutants. **(A–D)** Different developmental stage flowers **(A–C)** and carpel **(D)** of the wild-type. Sab, abaxial sepal; Sl, lateral sepal; Sad, adaxial sepal; Stp, inner antepetal stamen; Sts, outer antesepal stamen; C, carpel primordium; A, alae; K, keel; Vx, vexillum; Sg, stigma. Bars = 100 μm. **(E–H)** Different developmental stage of flowers **(E–G)** and carpel **(H)** in *mtaga* mutant. Red arrow points to the bulge structure. White arrows indicate trichomes. Bars = 100 μm. **(I–L)** Different developmental stage flowers **(I–K)** and carpel **(L)** of *mtagb* mutant. Blue arrow points to the petaloid structure of anther. Bars = 100 μm. **(M–P)** Different developmental stage flowers **(M–O)** and carpel **(P)** of *mtaga/+ mtagb-2/+*. Red arrow indicates a bulge at the base of carpel. Bars = 100 μm. **(Q–T)** Different developmental stage flowers **(Q–S)** and carpel **(T)** of *mtaga mtagb-2/+*. Yellow arrows point to split carpels, green arrow points to extra floral bud-like structure and white arrows indicate trichomes. Vx, vexillum. Bars = 100 μm. **(U–X)** Different developmental stage flowers **(U–W)** and carpel **(X)** of *mtaga/+ mtagb-2*. Green arrow points to extra floral bud-like structure at the carpel base. Vx, vexillum; K, keel. Bars = 100 μm. **(Y–A1)** Different developmental stage flowers of the *mtaga mtagb-2* double mutant. Asterisks indicate abnormal floral meristems which will develop to petals. Bars = 100 μm.

To further understand the effects of mutation of the duplicated *MtAGa* and *MtAGb* on early stage floral development in *M. truncatula*, we used SEM to compare flowers of *mtag* mutants with that of wild-type. All *mtag* mutants, except the homozygous *mtaga mtagb-2* double mutant, displayed similar floral ontogeny as wild-type (**Figures 7A,E,I,M,Q,U**). In the double mutant, the sepals were indistinguishable from that of wild-type flowers, but there were no obvious petals, stamens, as well as carpel primordia in the inner three whorls, and several FMs could be observed (**Figures 7A,Y**), which continued to generate vexillum-like petals as well as new FMs. Differences between *mtag* mutants and wild-type flowers became evident when the primordia of the third- and fourth-whorl organs started to differentiate (**Figures 7B–D,F–H,J–L,N–P,R–T,V–X,Z,A1**). In wild-type, the filaments and the anther locules appeared after stage 7 (Benloch et al., 2003) and then the central carpel became closed (**Figures 7B–D**). However, at the same stage, trichomes formed on the abaxial side of the carpel and bulges were observed at the base of the carpel margin in *mtaga* (**Figures 7F–H**). This observation may explain why mature *mtaga* flowers contain split carpels. Meanwhile, some weak petaloids in the anther tips were observed in *mtagb-2* flowers (**Figure 7K**). In the double heterozygote *mtaga/+ mtagb-2/+* plants, no obvious developmental defects exhibited in whorls 1-3 (**Figures 7M–O**), but there was one bulge at carpel base (**Figure 7P**).

Compared to wild type, the *mtaga mtagb-2/+* and *mtaga/+ mtagb-2* mutants displayed serious developmental defects in whorl 3 and 4. In *mtaga mtagb-2/+*, anthers were transformed into petaloid structures (**Figures 7R,S**) and the carpel clearly changed into compound leaf-like structure with trichomes and a floral bud-like organ was observed at its base (**Figures 7R–T**). Similarly, anthers also changed into petal-like structures in *mtaga/+ mtagb-2* (**Figures 7V,W**) and floral bud-like organs formed at the base of carpels (**Figure 7X**). In the *mtaga mtagb-2* double mutant, there were no gynoecium primordia formed but replaced by several meristem-like tissues, which continue to produce petal primordia (**Figures 7Z,A1**).

### The Expression Detection of Putative “ABCDE” Model Genes in *mtag* Mutants

To further understand the molecular basis of abnormal floral organs in diverse *mtag* mutants, we compared the expression of *M. truncatula* homologs of A, B, C, D, and E function genes in flowers of wild-type and different *mtag* mutants. First, we searched for potential A, B, D, and E function genes in *M. truncatula* database using the Arabidopsis A, B, D and E function proteins as a BLAST query in the National Center for Biotechnology Information (NCBI) and Phytozome (Supplementary Figure S6). Besides the previously identified *AP1*-like gene *MtPIM* (Benlloch et al., 2006), *PI* homologs *MtPI* and *MtNGL9* (Benlloch et al., 2009), *AP3*-like genes *MtNMH7* and *MtTM6* (Roque et al., 2013), *AG* homologs *MtAGa* and *MtAGb* (Serwatowska et al., 2014), and the *PLENA*-like gene *MtSHP* (Serwatowska et al., 2014), one additional *AP1*-like gene *Medtr5g046790* (named *MtAP1b*), two *AP2*-like genes *Medtr4g094868* and *Medtr5g016810* (named *MtAP2a* and *MtAP2b*, respectively), one *STK*-like gene *Medtr3g005530* (named *MtSTK*) and five *SEPALLATA*-like genes *Medtr6g015975, Medtr7g016600, Medtr3g084980, Medtr8g097090*, and *Medtr4g109810* (named, respectively, *MtSEP1/2a, MtSEP1/2b, MtSEP3a, MtSEP3b, MtSEP4*) were identified. Phylogenetic analysis showed that MtPIM and MtAP1b were closely related to Arabidopsis AP1. Amino acid sequence alignment indicated that MtPIM and MtAP1b have the highly conserved MADS-box and K-box (Vandenbussche et al., 2003) (Supplementary Figure S7). The MtAP2a and MtAP2b sequences are also very close to Arabidopsis AP2. Sequence alignment indicated that both sequences have the highly conserved YRG element and RAYD element that are characteristic of the subfamily (Supplementary Figure S8). The MtSTK shows highest level of sequence identity with the STK protein from Arabidopsis (75% amino acid identity). MtSTK contains the conserved MADS-box and K-box (Supplementary Figure S9). Amino acid sequence alignment of MtSEPs with homologs from Arabidopsis showed that all five MtSEPs have the conserved MADS-box, I region, K-box and C region (Supplementary Figure S10). Phylogenetic analysis of MtSEP1-5 with other MADS-box homologs showed that MtSEPs belong to the SEP-like clade specifically (Supplementary Figure S6).

**FIGURE 8:**
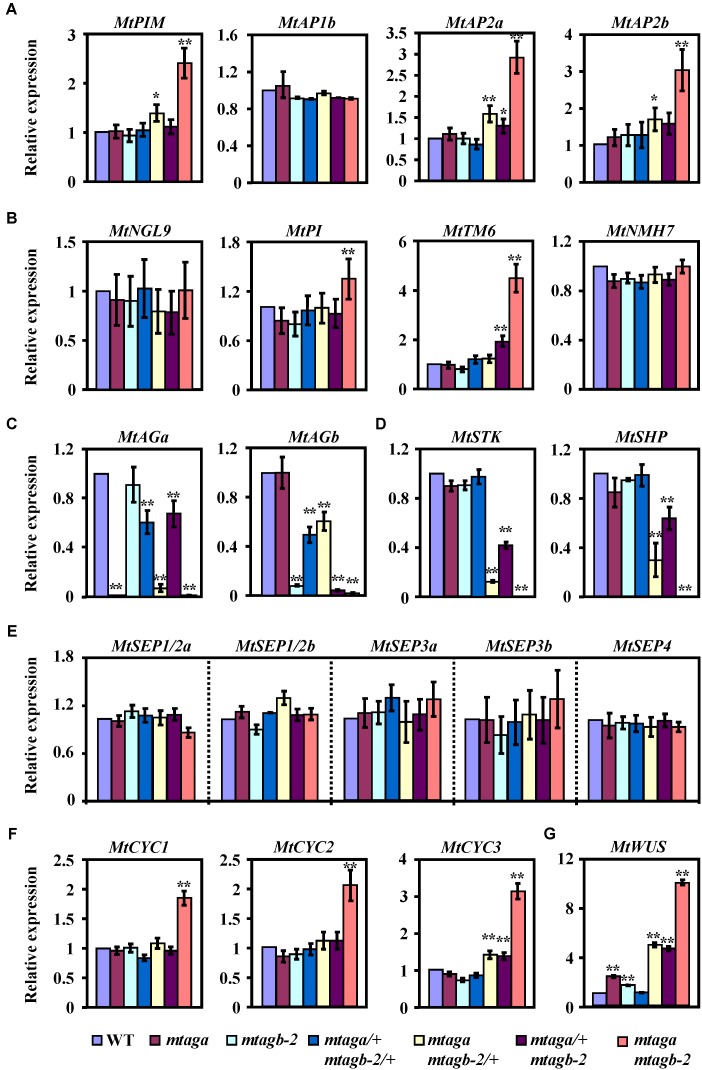
The expression levels of floral development related genes in the juvenile flowers of wild-type and *mtag* mutants. **(A)** Comparison of putative A function genes expression in the wild-type and different *mtag* mutants. **(B)** Expression of putative B function genes in the wild-type and different *mtag* mutants. **(C)** Expression of *MtAGa* and *MtAGb* in the wild-type and different *mtag* mutants. **(D)** Comparison of putative D function genes expression in the wild-type and different *mtag* mutants. **(E)** Expression of *M. truncatula SEP* homologs genes in the wild-type and different *mtag* mutants. **(F)** Comparison of TCP family genes *MtCYC1-3* expression in wild-type and different *mtag* mutants. **(G)** Expression of the *MtWUS* gene in wild-type and different *mtag* mutants. Values are means ± SE of three biological replicates. ^∗^*P* < 0.05, ^∗∗^*P* < 0.01 (Student’s *t*-test).

After identifying these putative *M. truncatula* “ABCDE” model homolog genes, we then analyzed their expression levels by qRT-PCR in juvenile flowers of different *mtag* mutants. We found that the expression of three putative A function genes *MtPIM, MtAP2a* and *MtAP2b*, was strongly upregulated in the *mtaga mtagb-2* double mutant but slightly upregulated in *mtaga mtagb-2/+*, while only *MtAP2a* was weakly upregulated in *mtaga/+ mtagb-2* (**Figure 8A**). This result coincides with the scenario that the A and C function genes negatively regulate each other. Loss of C function genes relieved the expression of A function genes (**Figures 8A,C**). Accordingly, the expression of two identified B function genes *MtPI* and *MtTM6*, which are required for petal and stamen identify, significantly increased in *mtaga mtagb-2*, and only *MtTM6* weakly increased in *mtaga/+ mtagb-2.* The expression of *MtNGL9* and *MtNMH7* was indistinguishable in wild-type and *mtag* mutants (**Figure 8B**). The expressions of these putative *M. truncatula* B function genes strongly diversified in *mtag* mutants, suggesting the existence of function differentiation. Consistent with the serious defects in ovule formation in *mtaga mtagb-2, mtaga mtagb-2/+* and *mtaga/+ mtagb-2* mutants, the expression of putative D function genes *MtSTK* and *MtSHP* was downregulated in above three mutants (**Figure 8D**). The decreased level of *MtSTK* and *MtSHP* appeared to be correlated with the different ovule defect phenotypes observed in *mtaga mtagb-2, mtaga mtagb-2/+* and *mtaga/+ mtagb-2* mutants, that is, *mtaga mtagb-2* had no ovule with the lowest *MtSTK* and *MtSHP* expression and *mtaga/+ mtagb-2* had the highest amount of ovule with the highest *MtSTK* and *MtSHP* expression. These results suggested that the ovule formation in *M. truncatula* is dependent on the level of *MtSTK* and *MtSHP* expression. However, compared to wild-type, the expression of five putative E function genes *MtSEP1/2a, MtSEP1/2b, MtSEP3a, MtSEP3b* and *MtSEP4* was not altered in all *mtag* mutants (**Figure 8E**), suggesting that loss of *MtAGs* does not affect the expression of *MtSEPs*, although *SEP* genes genetically interact with *AG* in controlling stamens and carpels identity.

### Expression Analysis of Floral Symmetry Genes in *mtag* Mutants

It has been reported that the TCP transcription factor CYCLOIDEA (CYC) is involved in the control of petal shape and floral zygomorphy in Fabaceae (Luo et al., 1996; Citerne et al., 2006; Feng et al., 2006; Wang et al., 2008; Xu et al., 2013). Because of presence of multiple vexillum-like petals in flowers of the *mtaga mtagb-2* double mutant (**Figure 5G**), we wondered whether this abnormal petal phenotype is related to the *CYC* genes and wanted to test the expression levels of the *M. truncatula* homologs of *CYC* in juvenile flowers of *mtag* mutants. Three potential CYC homologs (named here MtCYC1, MtCYC2, and MtCYC3) were found using the *Lotus japonicas* LjCYC1, LjCYC2 and LjCYC3 proteins as a BLAST query in the NCBI and Phytozome database. Phylogenetic analysis showed that MtCYC1, MtCYC2 and MtCYC3 are closely related to *L. japonicas* LjCYC1-3 (Supplementary Figure S11) and sequence alignment indicated that MtCYC1-3 sequences have the highly conserved TCP domain and R domain (Supplementary Figure S12), which are characteristic of the TCP transcription factors. qRT-PCR analysis revealed that the expression of *MtCYC1, MtCYC2* and *MtCYC3* were significantly upregulated in either the whole juvenile flowers or dissected petals of the *mtaga mtagb-2* double mutant, while only the expression of *MtCYC3* was slightly increased in *mtaga mtagb-2/+* and *mtaga/+ mtagb-2* (**Figure 8F** and Supplementary Figure S13). This result suggested that the abnormal dorsal petal shape observed in the *mtaga mtagb-2* double mutant might be associated with the ectopic expression of *MtCYC* genes. Taken together, our data demonstrated that the duplicated *AGAMOUS* homologs *MtAGa* and *MtAGb* redundantly fulfill a C-function activity in determining floral organ identity and coordinate with other floral homeotic genes and dorsoventral identity factors to control flower morphogenesis in *M. truncatula.*

## Discussion

The C function genes, belonging to the *AGAMOUS* (*AG*) lineage and encoding MADS-box transcription factors, are required for stamen and carpel identity and FM determinacy in angiosperms (Theissen, 2001; Theissen et al., 2016). Unlike Arabidopsis in which the *AGAMOUS* gene is represented by a single genomic sequence and essentially confers the C-function in the FM (Yanofsky et al., 1990; Wellmer et al., 2014), most extant legume plants are the products of an ancient genome duplication event and the presence of duplicated *AGAMOUS* homologs has been found in several legume species (Shoemaker et al., 2006; Serwatowska et al., 2014). Recent studies of the *AG* lineage genes in the model legume *M. truncatula* revealed that two *AG* homologs *MtAGa* and *MtAGb* are present in the *M. truncatula* genome and redundantly control the C-function activity in the third and fourth floral whorls during floral development (Serwatowska et al., 2014). In this study, we further investigated the genetic interaction of the duplicated *MtAG* homologs and uncovered interesting aspects of the regulatory control of floral organ identity as well as floral zygomorphy in *M. truncatula*.

### Conservation and Diversification of *MtAGa* and *MtAGb* Function

By characterizing the *MtAGa* and *MtAGb* loss-of-function insertional (*Tnt1*) mutants, we confirmed that both *MtAGa* and *MtAGb* shows a conserved C function activity, but with a little functional differentiation in determining the floral organ identity and FM determinacy. In consistent with previous description (Serwatowska et al., 2014), the *mtaga* mutants show partial conversion of stamens to petaloid structures, whereas serious defects in carpel fusion and ovule formation were observed at the early stage, leading to split carpels and producing abnormal pods with reduced seed number (**Figures 1**, **5B** and Supplementary Figures S1A,B). In contrast, the *mtagb* mutants produce flowers that display homeotic transformations predominantly in the third whorl (**Figures 3**, **5C**). Loss-of-function of *MtAGb* leads to an incomplete fusion of staminal tubes with weak stamen-petaloid conversion and occasionally resulting in split carpels (**Figures 3K,L**, **5C**, **6E**). These phenotypic differences between *mtaga* and *mtagb* mutants indicated that *MtAGa* may contribute more to carpel and ovule identity than *MtAGb*, whereas *MtAGb* appears to play a more important role in stamen identity than *MtAGa*. Besides, mutation of *MtAGa* causes extra structure formation at the base of carpels, which was not observed in the *mtagb* mutant (**Figures 1I,L**, **5B**), suggesting that *MtAGa* may play an important role in FM determinacy of *M. truncatula*.

The conserved function and subfunctionalization of *MtAGa* and *MtAGb* in controlling floral organ development may be partially explained by their spatial and temporal expression profile. qRT-PCR analysis revealed that both *MtAGa* and *MtAGb* predominantly express in floral tissues of stamens, carpels and ovules although *MtAGa* and *MtAGb* exhibit different expression levels (**Figures 4A,B**). In agreement with this result, previous northern blot and *in situ* hybridization analysis also indicated that the expression of both paralogs is distributed uniformly in whorls 3 and 4, while *MtAGb* shows a stronger signal than *MtAGa* (Serwatowska et al., 2014). Moreover, compared to the throughout expression of *MtAGa* in the whole FM, *MtAGb* transcript was observed in the region of the common primordia that will give rise to stamens at early development stage (Serwatowska et al., 2014). This specific expression is consistent with the notion that *MtAGa* and *MtAGb* are redundantly implicated in specification of the third and fourth whorls while *MtAGb* contributes more to stamen identity.

### Coordination of Floral Organ Identity Factors and Floral Zygomorphic Regulators in Controlling Flower Morphogenesis in *M. truncatula*

In Arabidopsis, *AGAMOUS* establishes the FM determinacy by repressing the key regulator of stem cell homeostasis *WUSCHEL* (Lenhard et al., 2001; Ming and Ma, 2009; Liu et al., 2011). Because *mtag* mutants not only show defects in stamen and carpel development but also exhibit serious indeterminate flower phenotypes, we wondered whether this would be the case in *M. truncatula* as well. qRT-PCR analysis showed that the transcript level of *MtWUS* (Chen et al., 2009) is significantly upregulated in all *mtag* mutants excepting the double heterozygous *mtaga/+ mtagb-2/+* (**Figure 8G**). Consistent with the FM termination determinacy phenotypes, the *mtaga mtagb-2* double mutant has the highest transcript levels of *MtWUS*, whereas the *mtaga mtagb-2/+* and *mtaga/+ mtagb-2* have intermediate levels, and the *mtaga* and *mtagb-2* single mutants have low levels of *MtWUS* transcript (**Figure 8G**). Although the function of *MtWUS* in meristem maintenance is unclear at this point, our data suggested that the molecular mechanism of FM determinacy through *AG*-mediated repression of *WUS* might be also conserved in *M. truncatula.*

In agreement with previous results that *MtAGa* and *MtAGb* show a C-function activity, the *mtaga mtagb-2* double mutant exhibits a complete loss of C function phenotype. The third whorl (stamens) and the fourth whorl (carpel) are entirely replaced by petals in the *mtaga mtagb-2* double mutant (**Figures 5G**, **6M**). However, in contrast to the single and double mutants, *mtaga mtagb-2/+* and *mtaga/+ mtagb-2* plants show an intermediate but distinct floral phenotypes (**Figures 5E,F**, **6I–L**), indicating a clear additive effect. Interestingly, despite the floral phenotypes of the double heterozygote *mtaga/+ mtagb-2/+* mutant are generally similar to that of wild-type, occasional split carpels were observed in *mtaga/+ mtagb-2/+*, suggesting a fine tuning expression of *MtAGa* and *MtAGb* in regulating carpel and stamen development. These results support the quartet model of floral organ specification (FQM) that floral homeotic gene function varies with respect to the amount of gene product required for different organ-specific tetrameric complexes (Theissen et al., 2016). The correlation between the level of *MtAGa* and *MtAGb* accumulation and the alteration of floral organ development suggests that the duplicated *AGAMOUS* homologs control *M. truncatula* C-function in a dose-depend manner.

In Arabidopsis, the A function genes (*AP1* and *AP2*) and C function gene (*AG*) antagonize each other, enforcing proper domains of activity (Bowman et al., 1989, 1991; Yanofsky et al., 1990). In consistency with this scenario, molecular analysis of *mtag* mutants revealed that the transcript levels of three putative A-function genes *MtPIM, MtAP2a* and *MtAP2b* are significantly upregulated in the *mtaga mtagb-2* double mutant (**Figure 8A**). Similarly, we also found that loss-of-function of *MtAGa* and *MtAGb* leads to significantly increased expression of a subset of floral homeotic B genes *MtPI* and *MtTM6* (**Figure 8B**), which is in agreement with previous report that floral homeotic C-function genes can repress the expression of specific B-function genes in California poppy (Yellina et al., 2010). These results indicated that this type of C-function-dependent regulation of A- and B- function genes may also be conserved in *M. truncatula.* In contrast to the upregulation of A- and B- function genes, the putative D-function genes *MtSTK* and *MtSHP* are significantly downregulated in *mtaga mtagb-2/+, mtaga/+ mtagb-2* and *mtaga mtagb-2* mutants (**Figure 8D**), which may explain the serious defects of ovule formation in above mutants. However, compared to wild-type, the expression of putative E function genes *MtSEP1-5* is not altered in the *mtaga mtagb-2* double mutant (**Figure 8E**), suggesting no direct regulation between *MtAGs* and *MtSEPs* at the transcriptional level. This is conceivable because the expression pattern of C function genes is not altered in the Arabidopsis *sep1 sep2 sep3* triple mutants as well, although the C and E function proteins form a protein complex postulated for stamen and carpel identity in FQM (Pelaz et al., 2000; Theissen et al., 2016).

It has been reported that *CYCLOIDEA* (*CYC*)-like TCP genes play key roles in dorsoventral differentiation of zygomorphic flowers in Papilionoideae legumes (Luo et al., 1996; Citerne et al., 2006; Feng et al., 2006; Wang et al., 2008; Xu et al., 2013). Considering the phenotype of multiple vexillum petals in flowers of the *mtaga mtagb-2* double mutant, we postulated that the expression of *M. truncatula* CYC homologs may be altered in *mtaga mtagb-2*. qRT-PCR analysis revealed that the expression of all three *MtCYC* homologs is significantly upregulated in the *mtaga mtagb-2* double mutant (**Figure 8F**), suggesting that the abnormal petal shape variation observed in *mtag* mutants may be caused by the ectopic expression of *MtCYC* genes. The hypothesis is further supported by findings in *Lotus japonicas* that ectopic expression of *CYC*-like genes *LjCYC1* and *LjCYC2* leads to the transformation of lateral and ventral petals to vexilla (Xu et al., 2013). These results indicated that loss-of-function of C function genes in *M. truncatula* may affect the flower dorsoventral differentiation by ectopically expressing *MtCYC* genes, although the direct/indirect regulation between *MtAG* and *MtCYC* genes remains to be elucidated. Further investigation of the genetic interaction between *MtAG* and *MtCYC* genes may add new insights into understanding the molecular mechanism underlying the floral morphogenesis in *M. truncatula.*

Taken together, our study analyze the functional conservation and diversification of the *M. truncatula* duplicated *AGAMOUS* homolog genes in regulating floral development and provide information for understanding the coordination of floral organ identity factors and floral dorsoventral identity regulators in determining flower morphogenesis of Papilionoideae legumes.

## Author Contributions

BZ, HuL, LN, and HaL designed the research. BZ, HuL, and LN performed the experiments. JW and KM contributed analytical tools. BZ, HuL, XW, YP, LN, and HaL analyzed the data. BZ, LN, and HaL wrote the manuscript.

## Conflict of Interest Statement

The authors declare that the research was conducted in the absence of any commercial or financial relationships that could be construed as a potential conflict of interest.

## Footnotes

1 http://www.ncbi.nlm.nih.gov/

2 https://phytozome.jgi.doe.gov/pz/portal.html

3 http://www.genome.jp/tools-bin/clustalw
