# Supplementary material for: Functional Specialization of Duplicated AGAMOUS Homologs in Regulating Floral Organ Development of Medicago truncatula
Source: Front Plant Sci. 2018 Jul 31;9:854. doi: 10.3389/fpls.2018.00854 (PMC6079578; doi:10.3389/fpls.2018.00854)
Supplement: Supplementary file 1 [file Table_1.PDF]

Table S1. Primers used in this study

Primers	Sequences 5'-3'	Application
NF19601-F	AAGCCGATACCTCATTACAC	For genotyping of NF19601
NF19601-R	TCTATCTCCTGCTGTGTACA	
NF10148-F	AAAGATTGGAAGGGGAAAGA	For genotyping of NF10148
NF10148-R	CGTCCACGGCTAGAGAAGAC	
NF13380-F	ATGGATTTTCCAAATGAATCC	For genotyping of NF13380
NF13380-R	TCCACGGCTAGAGAAGACT	
NF15934-F	ATGAGTTTTCCAAACGAATCC	For genotyping of NF15934
NF15934-R	GCATATTCATAGAGGCGGCC	
NF4908-F	GTCAAGTTACGTTCTGCAAG	For genotyping of NF4908
NF4908-R	AGTGAATCTTGGTTGCAGAA	
LTR6	GCTACCAACCAAACCAAGTCAA	Primers in Tnt1for genotyping of
LTR31	CTCCTCTCGGGGTCGTGGTT	different mutant
AGa-gateway-F	caccATGGATTTTCCAAATGAATC	For cloning of the *MtAGa* CDS and
AGa-gateway-R	TCAAACTAATTGAAGGGACA	RT-PCR
AGb-gateway-F	caccATGAGTTTTCCAAACGAATC	For cloning of the *MtAGb* CDS and
AGb-gateway-R	CACAAATTGAAGAGACATCT	RT-PCR
RT-MtActin-F	TCTTACTCTCAAGTACCCCATTGAGC	For RT-PCR analysis of *MtActin*
RT-MtActin-R	GTGGGAGTGCATAACCCTCATAGATT	
qMtActin-F	TCAATGTGCCTGCCATGTATGT	For qRT-PCR analysis of *MtActin*
qMtActin-R	ACTCACACCGTCACCAGAATCC	
qPCR-MtPIM-F	CAACTTTGAACATGGGTGGC	For qRT-PCR analysis of *MtPIM*
qPCR-MtPIM-R	TGGCAGGTATACAATGGTTCC	
qPCR-MtAP1b-F	TGTTGCACAAGAGGCTGC	For qRT-PCR analysis of *MtAP1b*
qPCR-MtAP1b-R	GGAAGAAGGGGATCTTGTAGTAAG	
qPCR-MtAP2a-F	TTTCCTATGGATCAAGAGTCTGATG	For qRT-PCR analysis of *MtAP2a*
qPCR-MtAP2a-R	CACGAGGAAAACTAGAAGAAGATG	
qPCR-MtAP2b-F	TCCGGTAGAGGATTCTGAGATG	For qRT-PCR analysis of *MtAP2b*
qPCR-MtAP2b-R	CACCTACCCAATGAGCTCTTG	
MtNGL9-qRTDIR	GTTATTTCAATTCCCAGATGCCA	For qRT-PCR analysis of *MtNGL9*
MtNGL9-qRTREV	GCTGCATAGGCTGAACACGAA	
MtPI-qRTDIR	CATATGGCACTTGAAGGTGTGG	For qRT-PCR analysis of *MtPI*
MtPI-qRTREV	TGGTTAAATCCATTGTCCATGC	
MtTM6-qRTDIR	CGAAGGAGACGAGGAATCAG	For qRT-PCR analysis of *MtTM6*
MtTM6-qRTREV	AGAGTGGAAGCACCATTGGC	
MtNMH7-qRTDIR	TTAGGTCCACGCATGTTTGC	For qRT-PCR analysis of *MtNMH7*
MtNMH7-qRTREV	AGGATTAGGATGAGTAGGCTGTAAGC	
qPCR-MtAGa-F	TCAAATGACTGCATTACAACCAAG	For qRT-PCR analysis of *MtAGa*
qPCR-MtAGa-R	CTAGGAAGCAAATTACAGGTCTTTC	
qPCR-MtAGb-F	CCAGGCAAGATCAGATGTCTC	For qRT-PCR analysis of *MtAGb*
qPCR-MtAGb-R	AGCCATCTGTTATCATAAGATTCCC	

qRT-MtSTK-F	GGGTGATGCATTAAGCACAC	For qRT-PCR analysis of *MtSTK*
qRT-MtSTK-R	TCCAGCTCAATCTCCCTTTTC	
qRT-MtSHP-F	GTGGAGTTCATGCAAAAGCG	For qRT-PCR analysis of *MtSHP*
qRT-MtSHP-R	ATGAAGGTAAGGACTGATCACAC	
qRT-MtSEP1/2a-F	AAATTGCCTTTTGGTTTTGACATTG	For qRT-PCR analysis of *MtSEP1/2a*
qRT-MtSEP1/2a-R	AAGTTTCAATAGTTTTAATTCAGTCACTG	
qRT-MtSEP1/2b-F	GATCAGATAGCTTCAACAAGTCAAG	For qRT-PCR analysis of *MtSEP1/2b*
qRT-MtSEP1/2b-R	CTTTTCCCTTTTCTGTAGCAAGC	
qRT-MtSEP3a-F	TCCAGGATCAGTGGTGGT	For qRT-PCR analysis of *MtSEP3a*
qRT-MtSEP3a-R	TTCACACTCTCTCTTTATATAGCTTTAATT	
qRT-MtSEP3b-F	CCATAGTGAAGCTCATGTTTCG	For qRT-PCR analysis of *MtSEP3b*
qRT-MtSEP3b-R	GTCAGTCCATTACATGTCAAAGG	
qRT-MtSEP4-F	GTTCCAGGTAGAATATTGGCTTTG	For qRT-PCR analysis of *MtSEP4*
qRT-MtSEP4-R	CGTCATAGCGTCGAATCCATTC	
qRT-MtCYC1-F	GAAGACGAAGGATTCTAGGGAG	For qRT-PCR analysis of *MtCYC1*
qRT-MtCYC1-R	CTCTTTGAAGTGATTTTGAAGTTTCATC	
qRT-MtCYC2-F	ATCTTCCACTAATCGCTCCAC	For qRT-PCR analysis of *MtCYC2*
qRT-MtCYC2-R	ATGTAGACGTGGATTGGTGC	
qRT-MtCYC3-F	CTGAAACTGAGGCTTATGCAAG	For qRT-PCR analysis of *MtCYC3*
qRT-MtCYC3-R	ATTCCTCAATCACATTAAATCCACC	
qRT-MtWUS-F	ACCTGAAATTGAAACCCTCCC	For qRT-PCR analysis of *MtWUS*
qRT-MtWUS-R	GCATCTTCAGCTTGATACCAAC	
