# Supplementary material for: Functional Specialization of Duplicated AGAMOUS Homologs in Regulating Floral Organ Development of Medicago truncatula
Source: Front Plant Sci. 2018 Jul 31;9:854. doi: 10.3389/fpls.2018.00854 (PMC6079578; doi:10.3389/fpls.2018.00854)
Supplement: Supplementary file 2 [file Table_2.PDF]

Table S2. Accession numbers used in this study

**Gene name and species**	**Accession numbers**
AP1 (Arabidopsis thaliana)	AT1G69120
AP2 (Arabidopsis thaliana)	AT4G36920
PI (Arabidopsis thaliana)	AT5G20240
AP3 (Arabidopsis thaliana)	AT3G54340
AG (Arabidopsis thaliana)	AT4G18960
STK (Arabidopsis thaliana)	AT4G09960
SHP1 (Arabidopsis thaliana)	AT3G58780
SHP2 (Arabidopsis thaliana)	AT2G42830
SEP1 (Arabidopsis thaliana)	AT5G15800
SEP2 (Arabidopsis thaliana)	AT3G02310
SEP3 (Arabidopsis thaliana)	AT1G24260
SEP4 (Arabidopsis thaliana)	AT2G03710
WUS (Arabidopsis thaliana)	AT2G17950
MtPIM (*Medicago truncatula*)	Medtr8g066260
MtAP1b (*Medicago truncatula*)	Medtr5g046790
MtAP2a (*Medicago truncatula*)	Medtr4g094868
MtAP2b(*Medicago truncatula*)	Medtr5g016810
MtPI (*Medicago truncatula*)	Medtr3g088615
MtNGL9 (*Medicago truncatula*)	Medtr1g029670
MtTM6 (*Medicago truncatula*)	Medtr5g021270
MtNMH7 (*Medicago truncatula*)	Medtr3g113030
MtAGa (*Medicago truncatula*)	Medtr2g017865
MtAGb (*Medicago truncatula*)	Medtr8g087860
MtSHP (*Medicago truncatula*)	Medtr3g452380
MtSTK (*Medicago truncatula*)	Medtr3g005530
MtSEP1/2a (*Medicago truncatula*)	Medtr6g015975
MtSEP1/2b (*Medicago truncatula*)	Medtr7g016600
MtSEP3a (*Medicago truncatula*)	Medtr3g084980
MtSEP3b (*Medicago truncatula*)	Medtr8g097090
MtSEP4 (*Medicago truncatula*)	Medtr4g109810
MtCYC1 (*Medicago truncatula*)	Medtr7g018500
MtCYC2 (*Medicago truncatula*)	Medtr6g017055
MtCYC3 (*Medicago truncatula*)	Medtr1g103380
MtWUS (*Medicago truncatula*)	Medtr5g021930
LjCYC1 (*Lotus japonicus*)	DQ202475
LjCYC2 (*Lotus japonicus*)	DQ202476
LjCYC3 (*Lotus japonicus*)	DQ202477
