# Supplementary material for: Functional Specialization of Duplicated AGAMOUS Homologs in Regulating Floral Organ Development of Medicago truncatula
Source: Front Plant Sci. 2018 Jul 31;9:854. doi: 10.3389/fpls.2018.00854 (PMC6079578; doi:10.3389/fpls.2018.00854)
Supplement: Supplementary file 3 [file Image_1.pdf]

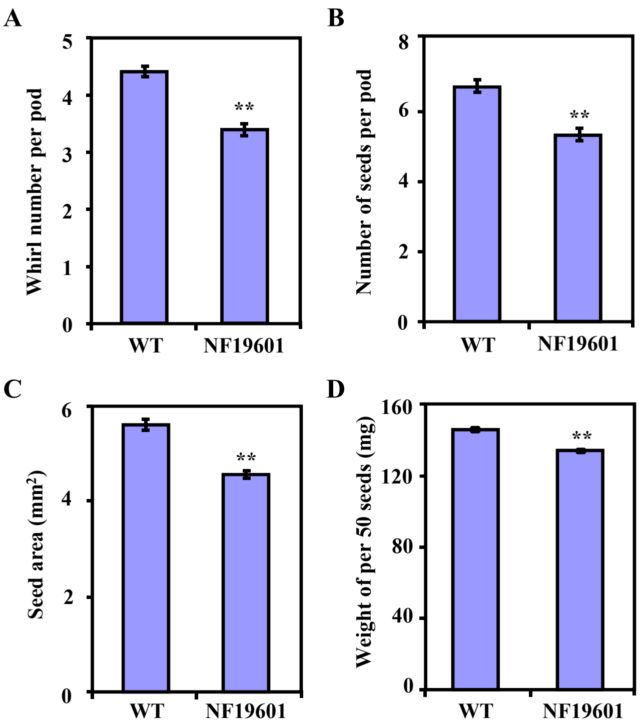

FIGURE S1. NF19601 mutant exhibits decreased pod and seed size. **(A)** Comparison of whirl number of the mature pod in the wild-type and NF19601. Values are means ± SE (n=30). ** P < 0.01 (Student's *t*-test). **(B)** Comparison of seed number per pod in the wild-type and NF19601. Values are means ± SE (n=30). ** P < 0.01 (Student's *t*-test). **(C)** Comparison of seed area in the wild-type and NF19601. Values are means ± SE (n=30). ** P < 0.01 (Student's *t*-test). **(D)** Comparison of seed weight in the wild-type and NF19601. Values are means ± SE (n=5). ** P < 0.01 (Student's *t*-test).
