# Supplementary material for: Functional Specialization of Duplicated AGAMOUS Homologs in Regulating Floral Organ Development of Medicago truncatula
Source: Front Plant Sci. 2018 Jul 31;9:854. doi: 10.3389/fpls.2018.00854 (PMC6079578; doi:10.3389/fpls.2018.00854)
Supplement: Supplementary file 4 [file Image_2.PDF]

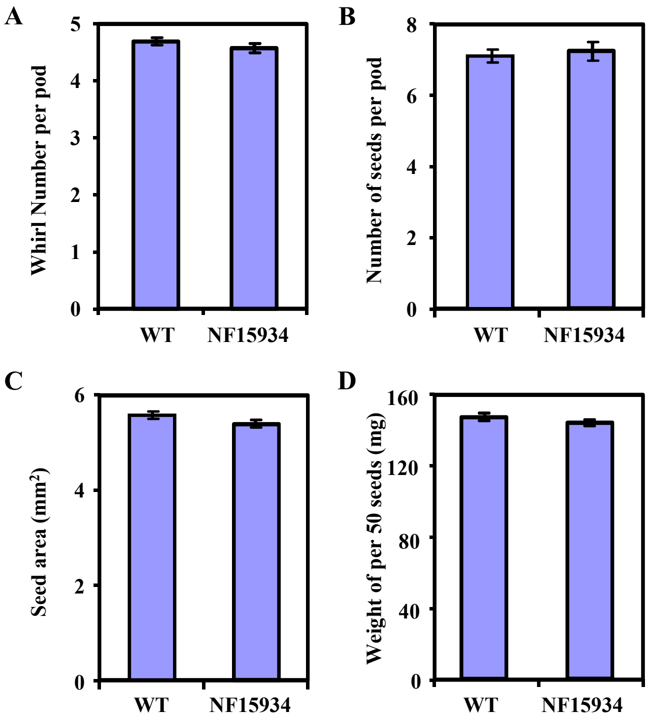

FIGURE S2. The pods and seeds of NF15934 are indistinguishable from that of the wild-type. **(A)** Comparison of whirl number of the mature pod in the wild-type and NF15934. Values are means ± SE (n=30). **(B)** Comparison of seed number per pod in the wild-type and NF15934. Values are means ± SE (n=30). **(C)** Comparison of seed area in the wild-type and NF15934. Values are means ± SE (n=30). **(D)** Comparison of seed weight in the wild-type and NF15934. Values are means ± SE (n=5).
