# Supplementary material for: Functional Specialization of Duplicated AGAMOUS Homologs in Regulating Floral Organ Development of Medicago truncatula
Source: Front Plant Sci. 2018 Jul 31;9:854. doi: 10.3389/fpls.2018.00854 (PMC6079578; doi:10.3389/fpls.2018.00854)
Supplement: Supplementary file 5 [file Image_3.PDF]

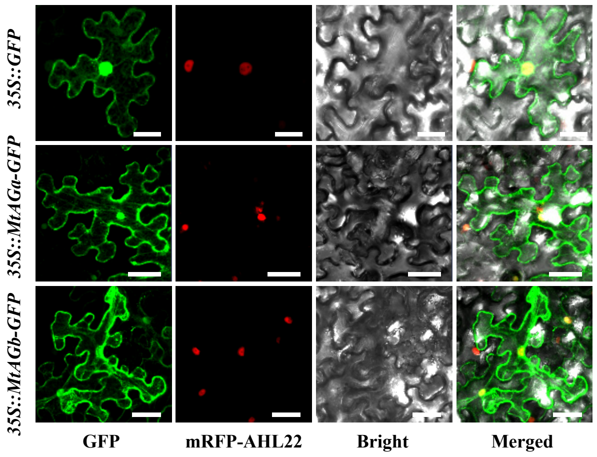

FIGURE S3. Subcellular localization of MtAGa and MtAGb. Subcellular localization of 35S::GFP (top panel), 35S::MtAGa-GFP (middle panel) and 35S::MtAGb-GFP (bottom panel) in *N. benthamiana* cells. Nuclear protein AHL22 was used as nuclear localization marker. Bars = 50 µm.
