# Supplementary material for: Functional Specialization of Duplicated AGAMOUS Homologs in Regulating Floral Organ Development of Medicago truncatula
Source: Front Plant Sci. 2018 Jul 31;9:854. doi: 10.3389/fpls.2018.00854 (PMC6079578; doi:10.3389/fpls.2018.00854)
Supplement: Supplementary file 6 [file Image_4.PDF]

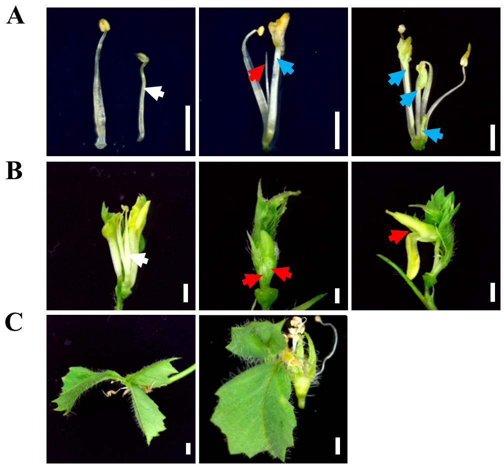

FIGURE S4. Phenotypes of *mtaga mtagb-2/+* in whorl 3 and whorl 4. **(A)** Dissected flowers of *mtaga mtagb-2/+* show extra stamen (white arrow), filament-like structure (red arrow), and petaloid tissues (blue arrows) in whorl 3. Bars = 1 mm. **(B)** Dissected flowers of *mtaga mtagb-2/+* show various stamen-like organs (white arrow), floral-bud like tissues (red arrows) in whorl 4. Bars = 1 mm. **(C)** The *mtaga mtagb-2/+* flowers usually yield leaves in stead of pods after flowering.
