# Supplementary material for: Functional Specialization of Duplicated AGAMOUS Homologs in Regulating Floral Organ Development of Medicago truncatula
Source: Front Plant Sci. 2018 Jul 31;9:854. doi: 10.3389/fpls.2018.00854 (PMC6079578; doi:10.3389/fpls.2018.00854)
Supplement: Supplementary file 7 [file Image_5.PDF]

A

B

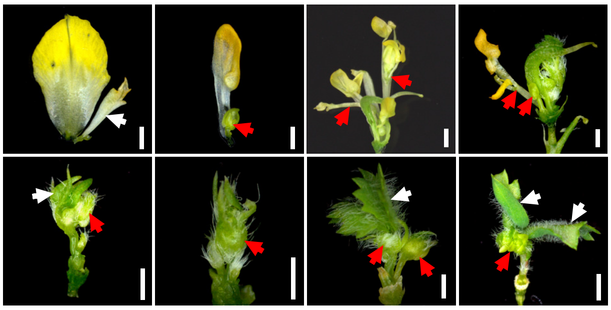

FIGURE S5. Phenotypes of *mtaga/+ mtagb-2* in whorl 3 and whorl 4. **(A)** Dissected flowers of *mtaga/+ mtagb-2* show extra petal (white arrow) and petaloid tissues (red arrows) in whorl 3. Bars = 1 mm. **(B)** Dissected flowers of *mtaga/+ mtagb-2* show various extra leaf-like (white arrows) and floral bud-like (red arrows) structures in the center of whorl 4. Carpels were removed. Bars = 1 mm.
