# Supplementary material for: Functional Specialization of Duplicated AGAMOUS Homologs in Regulating Floral Organ Development of Medicago truncatula
Source: Front Plant Sci. 2018 Jul 31;9:854. doi: 10.3389/fpls.2018.00854 (PMC6079578; doi:10.3389/fpls.2018.00854)
Supplement: Supplementary file 8 [file Image_6.PDF]

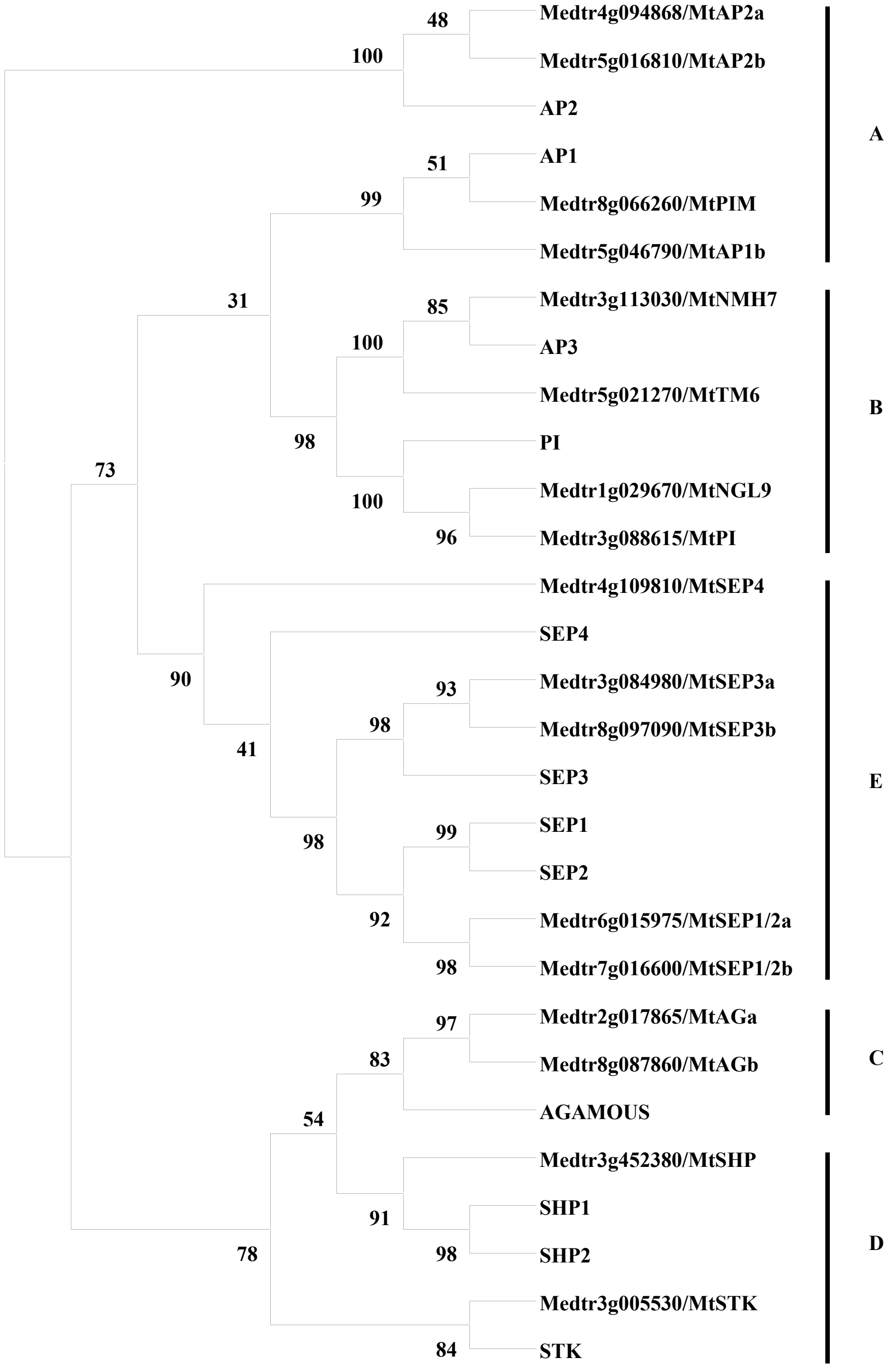

FIGURE S6. Phylogenetic analysis of putative "ABCDE" model members in *M. truncatula* and Arabidopsis. The putative *M. truncatula* "ABCDE" model member-like proteins group into five clades with the corresponding proteins from Arabidopsis. Numbers on branches indicate bootstrap values for 1000 replicates.
