# Supplementary material for: Functional Specialization of Duplicated AGAMOUS Homologs in Regulating Floral Organ Development of Medicago truncatula
Source: Front Plant Sci. 2018 Jul 31;9:854. doi: 10.3389/fpls.2018.00854 (PMC6079578; doi:10.3389/fpls.2018.00854)
Supplement: Supplementary file 9 [file Image_7.PDF]

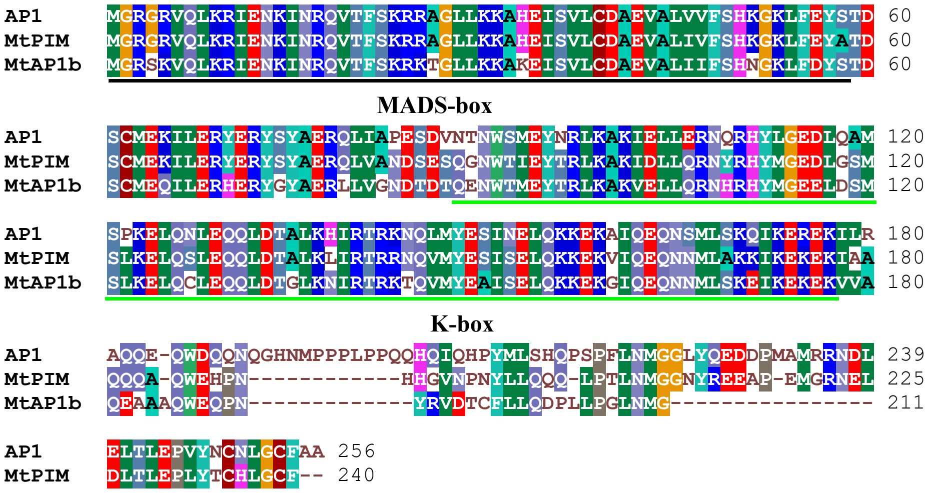

FIGURE S7. Amino acid sequence alignment of AP1, MtPIM and MtAP1b. The conserved MADS-box and K-box are underlined.
