# Supplementary material for: Functional Specialization of Duplicated AGAMOUS Homologs in Regulating Floral Organ Development of Medicago truncatula
Source: Front Plant Sci. 2018 Jul 31;9:854. doi: 10.3389/fpls.2018.00854 (PMC6079578; doi:10.3389/fpls.2018.00854)
Supplement: Supplementary file 12 [file Image_10.PDF]

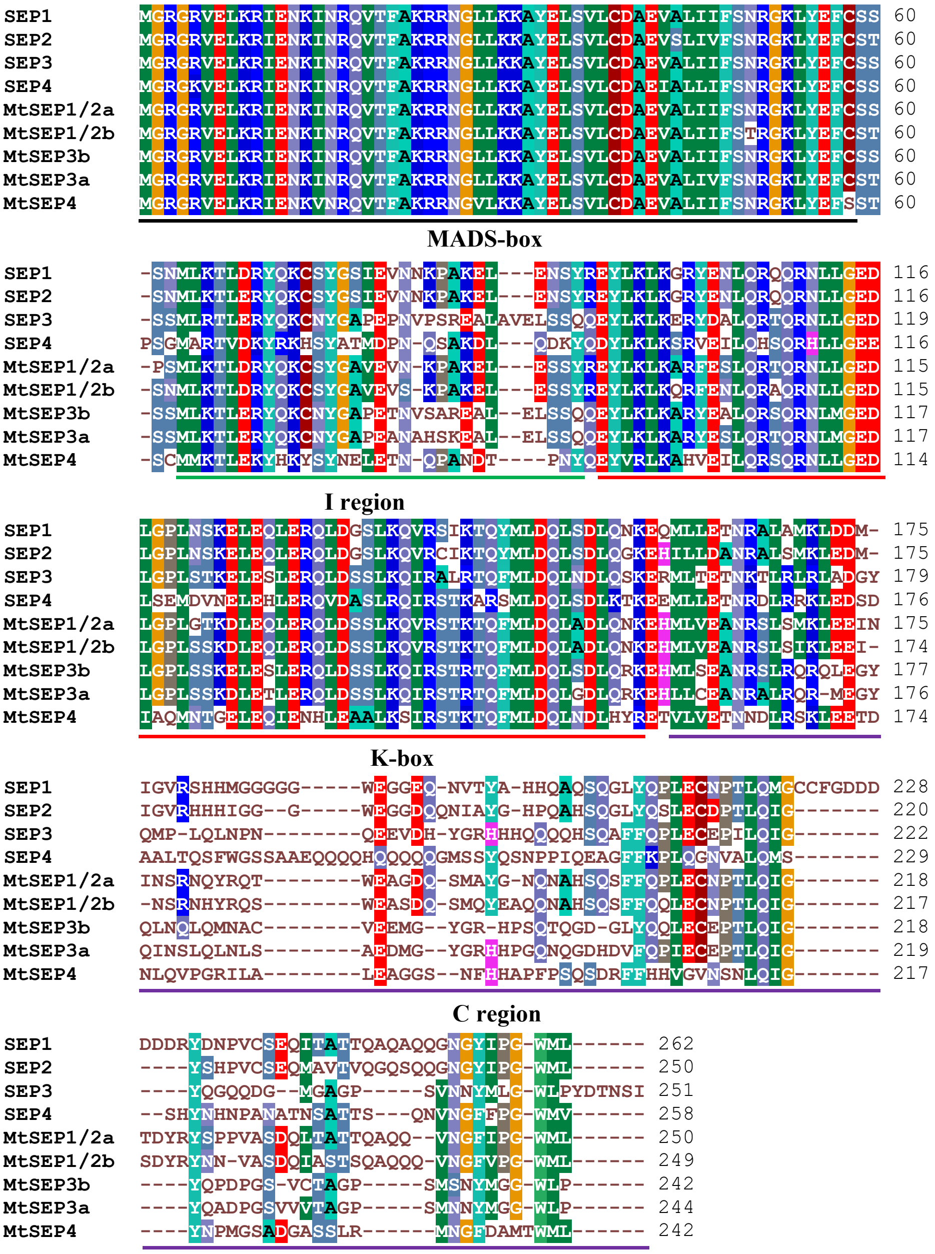

FIGURE S10. Sequence alignment of SEP proteins from *M. truncatula* and Arabidopsis. The conserved MADS-box, I region, K-box and C region are underlined.
