# Supplementary material for: Functional Specialization of Duplicated AGAMOUS Homologs in Regulating Floral Organ Development of Medicago truncatula
Source: Front Plant Sci. 2018 Jul 31;9:854. doi: 10.3389/fpls.2018.00854 (PMC6079578; doi:10.3389/fpls.2018.00854)
Supplement: Supplementary file 13 [file Image_11.PDF]

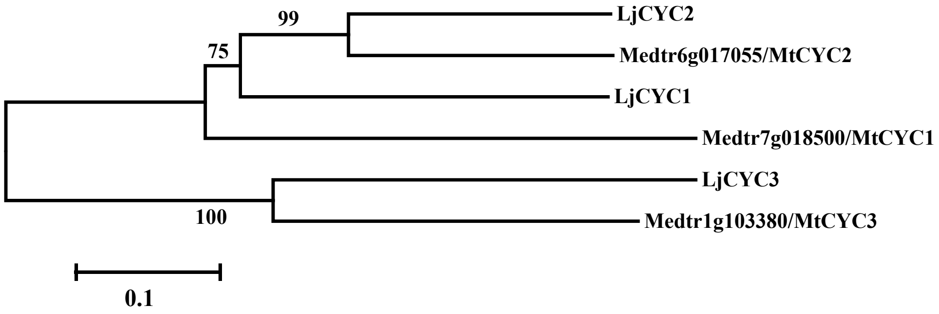

FIGURE S11. Phylogenetic analysis of CYC homologous proteins from *L. japonicas* and *M. truncatula*. Full-length amino acid sequences are aligned using ClustalW. Numbers on branches indicate bootstrap values for 1000 replicates.
