# Supplementary material for: Functional Specialization of Duplicated AGAMOUS Homologs in Regulating Floral Organ Development of Medicago truncatula
Source: Front Plant Sci. 2018 Jul 31;9:854. doi: 10.3389/fpls.2018.00854 (PMC6079578; doi:10.3389/fpls.2018.00854)
Supplement: Supplementary file 14 [file Image_12.PDF]

```
LjCYC1  ------------------------------------------------------------ 1
LjCYC2  ------------------------------------------------------------ 1
LjCYC3  ------------------------------------------------------------ 1
MtCYC1  ------------------------------------------------------------ 1
MtCYC2  ------------------------------------------------------------ 1
MtCYC3  MGVREERAFFGIEMKLCGRAFKKNTEFEVCITNLYYNFSLFRKMKMQFFLTELVYLRLSF 60

LjCYC1  -------------MFP---NSSYP------------YPYFP---SSSSSSPSPYPPFNFL 29
LjCYC2  -------------MFP---FSSSP------------YPSFP------SSSSSPFHPFSFL 26
LjCYC3  -------------MFSSTSYHDS-------------INPFP---SSFPSSSYP--SLPFL 29
MtCYC1  -------------MFPSTYCSLGP------------YPCYN---PNSSSSSHSYPPFTFL 32
MtCYC2  -------------MFP---YSSNP------------YPSFLPSSSSSSSSTSSLFPFPFL 32
MtCYC3  IQRRQYRSREATTVLNCDLFHTIPRVFNHWFQMFSFPNNFP---SSSSSSSYPNSTLSFL 117

LjCYC1  NPG-NAS-TNN--IFLHDPPPLP---SVPYTNNTTNTHHHHAPPTCSETFTNWALAD--- 79
LjCYC2  NPE-NVSASNN--TLVLDPLSVP---YIPQTHHH-HHHHHNNPPALPETLTNLAVAGSPD 79
LjCYC3  IDPENINNFPQD--PLVSHPFIH------------DSNIN-DAPIFTHDTTNLAVSVS-- 72
MtCYC1  TPDQNASSNTNNNNNNNNTNNINNFLHDPTISVPYTQTTHNHVPINPDTLTNWAVAD--- 89
MtCYC2  NPE-NASSNNNNNTLFHDPFSIP---YIPIHHHP-HPNLHN-ITNIPETLTNLAASQENN 86
MtCYC3  IDNPENNDFPNNNTTLLDDSLLS------------IPFTSTHHDVVPFPDTNFADCNN-- 163

LjCYC1  ------------AMLKQENLTGGPHHHYNLSNFLTR-------KLPAAKKPAKKDRHSKI 120
LjCYC2  N---------CFAMPKPDPS----GPHYGISCLLT-------------KRPAKKDRHSKI 113
LjCYC3  -----------HLLEQQDPLGGNNYASSIVPNFLRASKPAASASAAAAMATTKKDRHSKI 121
MtCYC1  ----------YAAMLKQDLS---GSSHYNLSNLLTK-------KP--VKKPAKKDRHSKI 127
MtCYC2  NNSNNNNNNIAIPMPKQDPSSLGGGSHYGISCFLA-------------KKPVKKDRHSKI 133
MtCYC3  -----------TAMFEQD----NGYNFGVTSSFPIPNFVVTEQKQPATVIGGKKDRHSKI 208

LjCYC1  HTSQGLRDRRVRLSIEIARKFFDLQDMLGFDKASNTLEWLFSKSNKAIEELFRSKHSDNN 180
LjCYC2  YTSQGLRDRRVRLSIEIARKFFDLQDMLGFDKARNTLEWLFNKSKRAIKDFARSK--NSN 171
LjCYC3  HTSQGLRDRRVRLSSEIARKFFDLQDMLEFDKPSNTLEWLFTKSESAIQELARSKNCSCS 181
MtCYC1  HTSQGLRDRRVRLSIEIARKFFDLQDMLGFDKASNTLEWLFNKSKEAIEELTRSK---NN 184
MtCYC2  YTSQGLRDRRVRLSIEIARKFFDLQDMLGFDKASNTLDWLFTKSKKAIKDLTKSKQRNNN 193
MtCYC3  HTSQGLRDRRVRLSSEIARKFFDLQDMLEFDKPSNTLEWLFTKSDTAIKELARTKNAACY 268
                           TCP domain

LjCYC1  NSTGA-----CAADGDGDGDDDSFSSS------------------SSEDEVVSVKRAQKE 217
LjCYC2  SGGGGDHDNNKSFSSEEDEDCEVVSSNLQQDQEQRLDSNHNKGGGAEKELLNKLKRAQ-- 229
LjCYC3  SNSNNN-------PSNNNKSLFGGGDNHN------------GTNSIRGRGNSKLKWTT-Q 221
MtCYC1  IASGD--------DDDGDHSFSSSSSD--------------EGDEEDYTKDRKMKRAQ-- 220
MtCYC2  SEVGG---DAKSFTSSSDCEDSEIATNNSSNLKQ-----HKEGAGSKHEEK-KLKRVQI- 243
MtCYC3  NNNNNDN--SPFFDSNSNKSFSGGGGGD-------------GSCSSKGR---KLKWTQ-K 309

LjCYC1  KEPSGVQA-KMKESREKARARARERTSN---------KMCPE-----------------T 250
LjCYC2  KEPSCARA-KIKESREKARARARERTSN---------KICNN-----------------N 262
LjCYC3  RDQDVCVLQNKKESRERARARARERTCY---------KML-------EDQRCP------A 259
MtCYC1  KESS-----KTKDSREKARARARERASEMKMQEDLKEKYPEI-----------------D 258
MtCYC2  KEPACVRASKMKESREKARARARERTSN---------KISNI-----------------R 277
MtCYC3  EE-------TKKESRERARARARERTCY---------KMCSSGRMQQEEIRYPGPGADST 353
                     R domain

LjCYC1  ENPQMQLHQFSSEF----------------QPHQELHR---------------------- 272
LjCYC2  NNTTLKKKLPATEN----------------SLILQQPKSPPRMLHHPHPHHLHHHLVGGE 306
LjCYC3  NTTQIMHQLRSSSP----------------IQLLQPY--PHLMDNSEAVSG------GGG 295
MtCYC1  QNQQILHQLMPNED----------------ETSKSLQR---------------------- 280
MtCYC2  GVQEVELKKKYENN----------------LQAFQQD-------HHS------------E 302
MtCYC3  NTQQMLQQLISSSTSPVDSTETEAYARWRQLLQLQTYSNPSFHNHHNLLDS------EIP 407

LjCYC1  --DDDFKVFEESIVIKRKLKQSLMSS---SHHHQ---NQNIGIPKEASFNNNNNSECPSS 324
LjCYC2  APRDDFNVIEESIVIKRKLKPWLMSSSSHHHHHQQQQHHNLVIPKEG--SFNNNSEYHSF 364
LjCYC3  GD--GFNVIEESIMIKRNMMSSNS-----HPHEN----HHLAIPNKE-AGFNNNYIDYPL 343
MtCYC1  --DDIFNFIEESIVIKRKLKK---SS---SHHYQ---QNTNLIPKEA--SFDHDS----- 322
MtCYC2  GPRNDFNVIEESIVIKRKLKPSMMVS---HHHHHPHYHQSHVIPKESSTSFNNNGDFNFL 359
MtCYC3  RDHGGFNVIEESIMIKRNMMSATSSQ---YHHQN----LIPTIPKEL-PSFNHNINDYSL 459

LjCYC1  FP---MSPNWD-----ASGATPRSNICAIASVNLSTGLQIFGKSWEEC-TSPHLH 370
LjCYC2  PN---SSPNWD--NNTSNSATSRSNFCTIASMNLSTGLQIFGKSWEEC-TNPRPH 413
LjCYC3  LQPYSTTPNWE----------IAT-----MNMNLST---CFMNQW---------- 370
MtCYC1  PM---LSPNWDANNNNNDAATGRSNFSAISRMNLSSGLQIFGKSWEDCNTSPNRY 374
MtCYC2  PN---SSPNWDA-NNNGTSSTNRSTFCAIASMNLSTGLQIFGKSWEEC-TNPRLH 409
MtCYC3  FP--CSTPNWE----------TSSNFCGIATMNLST---CFLNPW---------- 489
```

FIGURE S12. Amino acid sequence alignment of CYC homologues from *L. japonicas* and *M. truncatula.* The conserved TCP and R domains are underlined.
