# Supplementary material for: Functional Specialization of Duplicated AGAMOUS Homologs in Regulating Floral Organ Development of Medicago truncatula
Source: Front Plant Sci. 2018 Jul 31;9:854. doi: 10.3389/fpls.2018.00854 (PMC6079578; doi:10.3389/fpls.2018.00854)
Supplement: Supplementary file 15 [file Image_13.pdf]

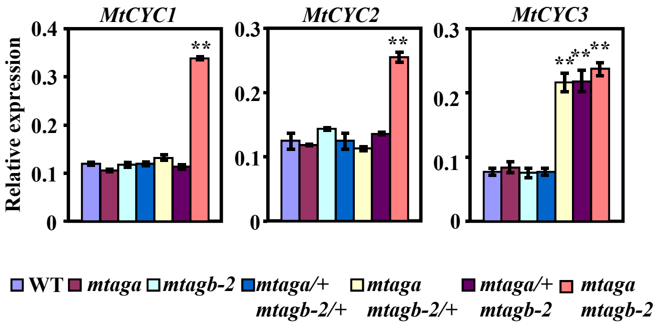

FIGURE S13. Comparison of TCP family genes *MtCYC1-3* expression in petals of wild-type and different *mtag* mutants. Values are means ± SE. ** P < 0.01 (Student's *t*-test).
